# TDRD3, a Tudor domain-containing protein, regulates *Klf2*-dependent T_reg_ differentiation and function to modulate immune tolerance

**DOI:** 10.1126/sciadv.aea3960

**Published:** 2026-01-23

**Authors:** Yun Shi, Xiaoqun Tao, Lei Shen, Yate-Ching Yuan, Guanpeng Wang, Ethan Eurmsirilerd, Rendell Chang, Zhenyu Jia, Weirong Shang, Yanzhong Yang, Zuoming Sun

**Affiliations:** ^1^ Department of Immunology & Theranostics, Arthur Riggs Diabetes & Metabolism Research Institute, Beckman Research Institute of the City of Hope, Duarte, CA 91010, USA.; ^2^ Department of Cancer Genetics and Epigenetics, Beckman Research Institute, City of Hope Cancer Center, Duarte, CA 91010, USA.; ^3^ Translational Bioinformatics, Department of Computational Quantitative Medicine, Beckman Research Institute of the City of Hope, Duarte, CA 91010, USA.; ^4^ Department of Botany & Plant Sciences, University of California, Riverside, CA 92527, USA.; ^5^ Department of Gynecology and Obsterics, School of Medicine, Emory University, Atlanta, GA 30322, USA.

## Abstract

Tudor domain-containing protein 3 (TDRD3) functions as a methylarginine reader that relays posttranslational arginine methylation signals to the transcriptional machinery, thereby regulating cellular functions through modulation of gene expression. Regulatory T cells (T_regs_) are pivotal for establishing and maintaining immune tolerance. In this study, we demonstrate that mice with T_reg_-specific deletion of *Tdrd3* exhibit severely impaired iT_reg_, but not thymic T_reg_, differentiation. Moreover, iT_regs_, but not thymic T_regs_, derived from these mice fail to suppress colitis in adoptive transfer models, indicating compromised inhibitory capacity. Aged *Tdrd3*-deficient mice also show spontaneous autoinflammation. Mechanistically, TDRD3 is recruited by the transcription factor FOXO1, presumably in a methylation-dependent manner, to activate *Klf2* expression, which is essential for T_reg_ differentiation. Notably, the enforced expression of *Klf2* in *Tdrd3*-deficient CD4^+^ T cells rescue both iT_reg_ development and suppressive function. Collectively, our findings identify TDRD3 as a central transcriptional regulator of iT_reg_ differentiation and immune homeostasis, highlighting it as a potential therapeutic target for modulating immune tolerance.

## Teaser

TDRD3 regulates immune tolerance via activating *Klf2* gene to promote iT_reg_ differentiation and function.

## INTRODUCTION

Regulatory T (T_reg_) cells are a specialized subset of CD4^+^ T lymphocytes that play a central role in establishing immune tolerance by preventing autoimmunity, maintaining immune homeostasis, and resolving immune responses after infection clearance (*1*, *2*). The lineage-specific transcription factor (forkhead box protein 3) Foxp3 is essential for the development and suppressive function of T_regs_, as demonstrated by the severe and uncontrolled autoinflammatory responses observed in both mice and humans harboring Foxp3 mutations that result in a complete loss of functional T_regs_ and failure to establish peripheral immune tolerance (*3*–*5*). Upon antigen stimulation, naïve CD4^+^ T cells undergo proliferation and differentiate into inflammatory effector T cells—including T helper 1 (T_H_1), T_H_2, and T_H_17 cells (*6*–*8*)—or are induced to become regulatory T cells [induced T_regs_ (iT_regs_) or peripheral T_reg_ (pT_regs_)] in the presence of transforming growth factor–β (TGF-β) (*9*, *10*), representing two functionally opposing subsets of helper T cells. A delicate balance between these opposing lineages is essential for maintaining immune system function. In contrast to iT_regs_, thymic T_regs_ are not induced but developed in the thymus mostly with T cell receptors (TCRs) recognizing self-antigens (*11*, *12*). Since it is difficult to obtain a large number of thymic T_regs_, the induction of inhibitory iT_regs_ from naïve T cell precursors represents a promising therapeutic strategy for autoimmune diseases. Therefore, elucidating the mechanisms that regulate this conversion process is important.

Arginine methylation is a posttranslational modification that plays critical roles in regulating diverse biological processes, including transcription and signal transduction (*13*, *14*). The mammalian genome encodes ~30 Tudor domain-containing proteins, which recognize and bind methylated arginine or lysine residues on target proteins. These interactions serve to transmit posttranslational modification signals to the transcriptional machinery, thereby modulating gene expression (*15*–*17*). Tudor domain-containing protein 3 (TDRD3) was identified over three decades ago as the first reader of methylarginine marks (*18*) and has since been shown to play essential roles in transcriptional regulation and genome stability (*19*–*21*). TDRD3 contains three conserved domains: an N-terminal oligonucleotide-binding (OB) fold domain, a ubiquitin-associated (UBA) domain, and a C-terminal Tudor domain (*22*, *23*). The Tudor domain is responsible for recognizing methylarginine residues deposited by protein arginine methyltransferases. Genome-wide analyses have shown that TDRD3 predominantly binds regions immediately upstream of transcriptional start sites (*18*, *19*, *21*, *24*), supporting its role as a transcriptional coregulator. Although *Tdrd3* knockout (*Tdrd3*^−/−^) mice are viable, fertile, and develop normally (*19*), *Tdrd3*-deficient mouse embryonic fibroblasts (MEFs) exhibit increased DNA damage, and B cells from these mice show elevated levels of chromosomal translocations (*19*), suggesting tissue-specific functions for TDRD3.

Here, we report a critical role for *Tdrd3* in the differentiation and suppressive function of iT_regs_, but not thymic T_regs_. Naïve CD4^+^ T cells from T_reg_-specific *Tdrd3*-deficient mice (*Tdrd3^fl/fl^/Foxp3^YFP-Cre^*) exhibited impaired iT_reg_ differentiation both in vitro and in vivo. Consistently, aged *Tdrd3^fl/fl^/Foxp3^YFP-Cre^* mice developed spontaneous autoinflammatory symptoms, including splenomegaly, weight loss, and lymphocytic infiltration in the lungs and liver, with CD4^+^ T cells producing elevated levels of inflammatory IFN-γ. Furthermore, these mice displayed exacerbated symptoms in the experimental autoimmune encephalomyelitis (EAE) model, associated with a reduction in T_reg_ frequency and an increase in inflammatory CD4^+^ T cells in the central nervous system. iT_regs_ derived from *Tdrd3^fl/fl^/Foxp3^YFP-Cre^* mice also exhibited impaired suppressive function, both in vitro, in inhibiting CD4^+^ T cell proliferation, and in vivo, in an adoptive transfer model of colitis. Mechanistically, TDRD3 is recruited by FOXO1, presumably in a methylation-dependent manner, to activate expression of *Klf2*, a transcription factor critical for T_reg_ differentiation and function (*25*, *26*). Forced expression of *Klf2* in *Tdrd3*-deficient CD4^+^ T cells restored both iT_reg_ differentiation and their suppressive function. Together, these findings highlight a vital role for TDRD3 in maintaining immune tolerance through the regulation of iT_reg_ differentiation and function.

## RESULTS

### TDRD3 is dispensable for thymic T_reg_ development but plays an essential role in the T_regs_ differentiation

**Fig. 1.**
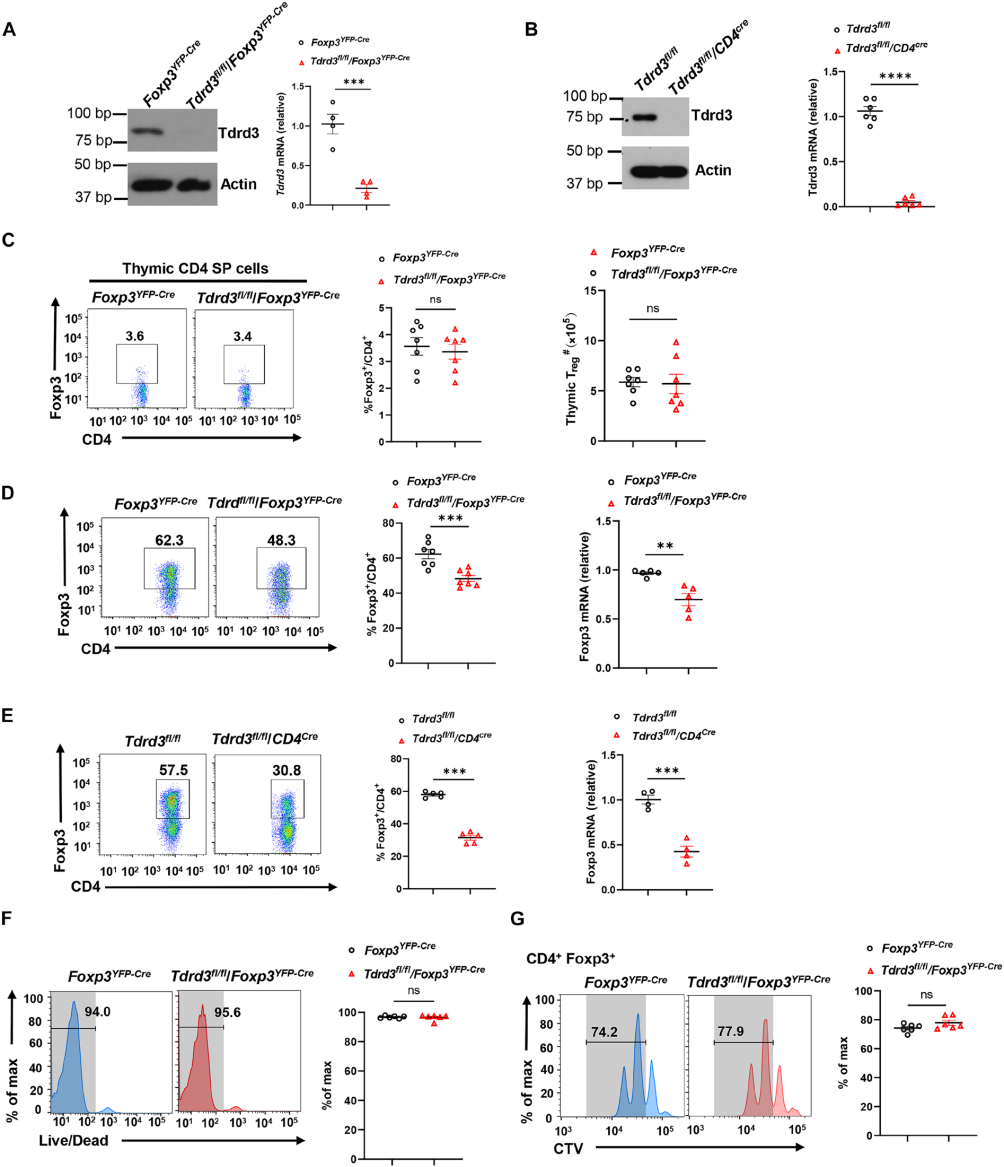
TDRD3 is dispensable for thymic T_reg_ development but plays an essential role in the differentiation of regulatory T cells from naïve CD4^+^ T cells. (**A** and **B**) Immunoblot analysis of TDRD3 in CD4^+^YFP^+^ T_regs_ (A) or CD4^+^ cells (B) isolated from the spleen of indicated mice. Right: qPCR analysis of *Tdrd3* mRNA (*n* ≥ 3). (**C**) Representative flow cytometric analysis (left two panels), percentage (third panel) and number (right panel) of T_reg_ (Foxp3^+^) cells in thymocytes from indicated mice (*n* ≥ 6). (**D** and **E**) Representative flow cytometric analysis (left two panels) and percentage (third panel) of Foxp3^+^ iT_regs_ among CD4^+^ T cells from *Foxp3^YFP-Cre^* or *Tdrd3^fl/fl^/Foxp3^YFP-Cre^* mice (D) or from *Tdrd3^fl/fl^* and *Tdrd3^fl/fl^/CD4^Cre^* mice (E), polarized in the presence of TGF-β (5 ng/ml) for 48 hours (*n* ≥ 6 per treatment cohort). Foxp3 mRNA levels in differentiated iT_regs_ were detected by qPCR (right). (**F**) Representative flow cytometric analysis (two left panels) and percentage (right panel) of live cells among Foxp3^+^ iT_regs_ differentiated from naïve CD4^+^ T cells of indicated mice (*n* ≥ 6). (**G**) Representative flow cytometric analysis (two left panels) and percentage (right panel) of the proliferating *Foxp3^YFP-Cre^* or *Tdrd3^fl/fl^/Foxp3^YFP-Cre^* CD4^+^ T cells in indicated peaks shown on left, labeled with CellTrace Violet (CTV) and polarized under T_reg_ conditions for 48 hours (*n* ≥ 6). Boxed area: cell population of interest. Data are from three independent experiments [(A), (B), (C), (E), (F), and (G), right panels; presented as means ±SEM] or are from one representative of three independent experiments [(A), (B), (C), (E), (F), and (G), left panels]. **P < 0.01, ***P < 0.001, and ****P < 0.0005; ns, not significant (two-tailed Student’s t test).

To dissect TDRD3 function in T_regs_, we generated a strain of T_reg_-specific knockout mouse model, *Tdrd3^fl/fl^/Foxp3^YFP-Cre^*. In addition, we occasionally used *Tdrd3^fl/fl^/CD4^Cre^* mice that delete *Tdrd3* in T cells. The deletion of *Tdrd3* in T_regs_ and CD4^+^ T cells was confirmed by both immunoblot (Fig. 1, A and B, left panels) and quantitative polymerase chain reaction (qPCR) analysis of *Tdrd3* expression (Fig. 1, A and B, right panels).

We first examined the impact of *Tdrd3* deletion on thymic T_reg_ development. The overall thymocyte development was unaltered in *Tdrd3^fl/fl^/Foxp3^YFP-Cre^* mice, as indicated by normal thymic cellularity (fig. S1A) and comparable frequencies of major thymocyte subsets, including CD4^−^CD8^−^ double-negative (DN), CD4^+^CD8^+^ double-positive (DP), and CD4^+^ or CD8^+^ single-positive (SP) cells, relative to *Foxp3^YFP-Cre^* controls (fig. S1B). Both the percentage and absolute number of thymic T_regs_ were similar between *Tdrd3^fl/fl^/Foxp3^YFP-Cre^* and *Foxp3^YFP-Cre^* mice (Fig. 1C), indicating that TDRD3 is not required for thymic T_reg_ development.

Next, we assessed TDRD3’s role in iT_reg_ differentiation from naïve CD4^+^ T cells. Purified naïve CD4^+^ T cells were negative for both yellow fluorescent protein–positive (YFP^+^) and Foxp3^+^, confirming lack of T_regs_ (fig. S1C). In addition, differentiated iT_reg_ were positive for both YFP and Foxp3, confirming that YFP represents Foxp3 expression (fig. S1D). Under TGF-β–induced conditions, naïve CD4^+^ T cells from *Tdrd3^fl/fl^*/*Foxp3^YFP-Cre^* mice showed substantially impaired iT_reg_ differentiation, as indicated by reduced frequencies of Foxp3^+^ (Fig. 1D, three left panels and fig. S1E in which YFP was used for monitoring iT_regs_) compared to *Foxp3^YFP-Cre^* mice controls. Consistently, *Foxp3* mRNA levels were also markedly reduced in iT_regs_ derived from *Tdrd3^fl/fl^*/*Foxp3^YFP-Cre^* CD4^+^ T cells (Fig. 1D, right). Similar defective iT_reg_ differentiation and lower *Foxp3* mRNA levels were also observed when naïve CD4^+^ T cells from *Tdrd3^fl/fl^*/*CD4^Cre^* mice were used for iT_reg_ differentiation (Fig. 1E), confirming the essential role for TDRD3 in iT_reg_ differentiation. The impaired iT_reg_ differentiation was not due to differences in cell survival (Fig. 1F) or proliferation (Fig. 1G), both of which remained comparable between *Tdrd3^fl/fl^/Foxp3^YFP-Cre^* and *Foxp3^YFP-Cre^* CD4^+^ T cells under T_reg_-polarizing conditions.

Together, these findings demonstrate that while TDRD3 is not required for thymic T_reg_ development, it is critically required for iT_reg_ differentiation from naïve CD4^+^ T cells, highlighting a distinct function in these two types of T_regs_.

### TDRD3 is critical for iT_reg_ differentiation and immune regulation in vivo

**Fig. 2.**
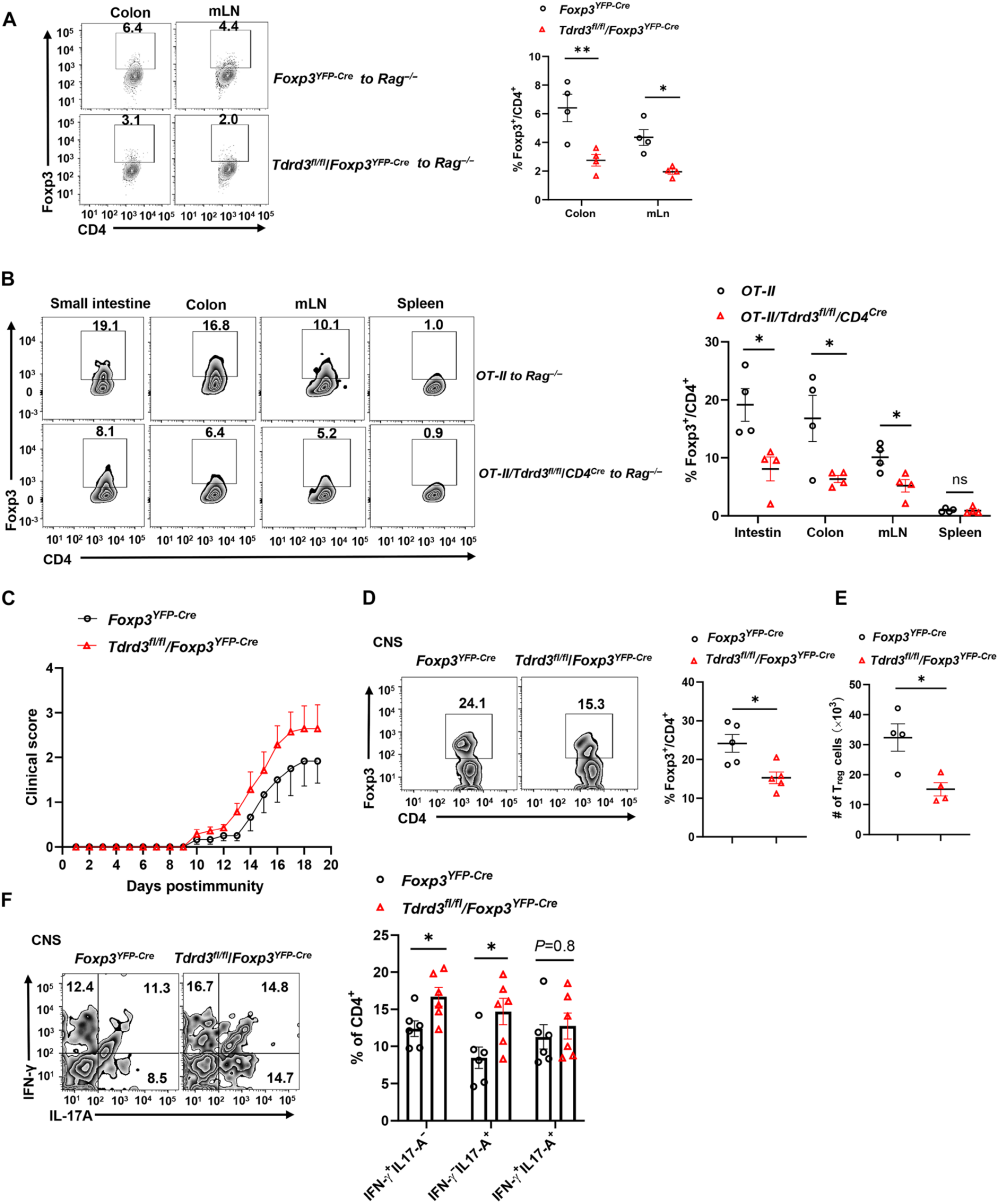
TDRD3 is critical for iT_reg_ differentiation and immune regulation in vivo. (**A**) Representative flow cytometric analysis and percentage of Foxp3^+^CD4^+^ T_regs_ in colon and mLNs 3 weeks after adoptive transfer of 0.4 × 10^6^ naïve CD4^+^ cells from indicated mice to *Rag1^−/−^* recipients (*n* ≥ 4). (**B**) Representative flow cytometric analysis and percentage of Foxp3^+^CD4^+^ T_regs_ in small intestine, colon, mLN, and spleen of *Rag1^−/−^* mice adoptively transferred with 3 × 10^6^ CD4^+^ T cells from *OT-II* or *OT-II/Tdrd3^fl/fl^/CD4^Cre^* mice and subsequently fed with OVA-containing drinking water (20 mg/ml) for 10 days (*n* ≥ 4). (**C**) Mean clinical EAE scores of indicated mice at different days after EAE induction with MOG_35–55_ immunization (*n* ≥ 6). (**D** and **E**) Representative flow cytometric analysis [(D), left two panels], the percentage [(D), right], and number (E) of Foxp3^+^CD4^+^ T_regs_ recovered from the CNS of EAE-induced mice shown in C (*n* ≥ 5). (**F**) Representative flow cytometric analysis (left two panels) and percentage (right) of IFN-γ^+^, IL-17A^+^, and IFN-γ^+^ IL-17A^+^cells among CD4^+^ T cells recovered from the CNS of EAE-induced mice shown in C (*n* ≥ 6). Boxed area: cell population of interest. Data are from three experiments [(C) and (E); (A), (B), (D), and (F), right panels, presented as means ± SEM] or are from one representative of three independent experiments [(A), (B), (D), and (F), left panels]. *P < 0.05, **P < 0.01 (two-tailed Student’s *t* test). CNS, central nervous system.

To evaluate the in vivo role of TDRD3 in iT_reg_ differentiation, isolated naïve CD4^+^ T cells, lack of YFP^+^ T_regs_ (fig. S1C), from *Tdrd3^fl/fl^/Foxp3^YFP-Cre^* or *Foxp3^YFP-Cre^* mice, were adoptively transferred into *Rag1^−/−^* recipients. Three weeks posttransfer, substantial numbers of Foxp3^+^ T_regs_ were detected in the mesenteric lymph nodes (mLNs) and colon (Fig. 2A). iT_regs_ derived from *Tdrd3^fl/fl^*/*Foxp3^YFP-Cre^* CD4^+^ T cells were markedly reduced compared to those from control CD4^+^ T cells (Fig. 2A).

We next used an oral tolerance model to assess the impact of TDRD3 on antigen-specific iT_reg_ induction. Naïve CD4^+^ T cells from *OT-II* or *OT-II*/*Tdrd3^fl/fl^/CD4^Cre^* mice were transferred into *Rag1^−/−^* mice, followed by oral administration of ovalbumin (OVA) peptide via drinking water (Fig. 2B). T_regs_ were efficiently induced in the gut-associated lymphoid tissues of mice receiving OT-II CD4^+^ T cells. In contrast, mice receiving CD4^+^ cells from *OT-II/Tdrd3^fl/fl^/CD4^Cre^* mice exhibited a markedly reduced frequency of T_regs_ in the small intestine, colon, and mLN. No difference in T_regs_ was observed in the spleen, which is not involved in gut-associated tolerance (Fig. 2B). These findings establish that TDRD3 is required for efficient antigen-specific iT_reg_ differentiation in gut lymphoid tissues in vivo.

To determine whether impaired T_reg_ generation due to TDRD3 deficiency contributes to autoimmune pathology, we induced EAE, a model of central nervous system autoimmunity. Compared to *Foxp3^YFP-Cre^* controls, *Tdrd3^fl/fl^*/*Foxp3^YFP-Cre^* mice developed more severe EAE symptoms (Fig. 2C). This was accompanied by reduced percentages and numbers of T_regs_ in the central nervous system (Fig. 2, D and E), and increased frequencies of proinflammatory interferon-γ (IFN-γ)– and interleukin-17A (IL-17A)–producing CD4^+^ T cells (Fig. 2F and gating strategy in fig. S2A). Together, these results demonstrate that TDRD3 is essential for iT_reg_ generation in vivo and that TDRD3-regulated T_reg_ differentiation is critical for controlling the magnitude of immune responses and maintaining immune tolerance.

### TDRD3 is required for the suppressive function of iT_regs_ but not thymic T_regs_

**Fig. 3.**
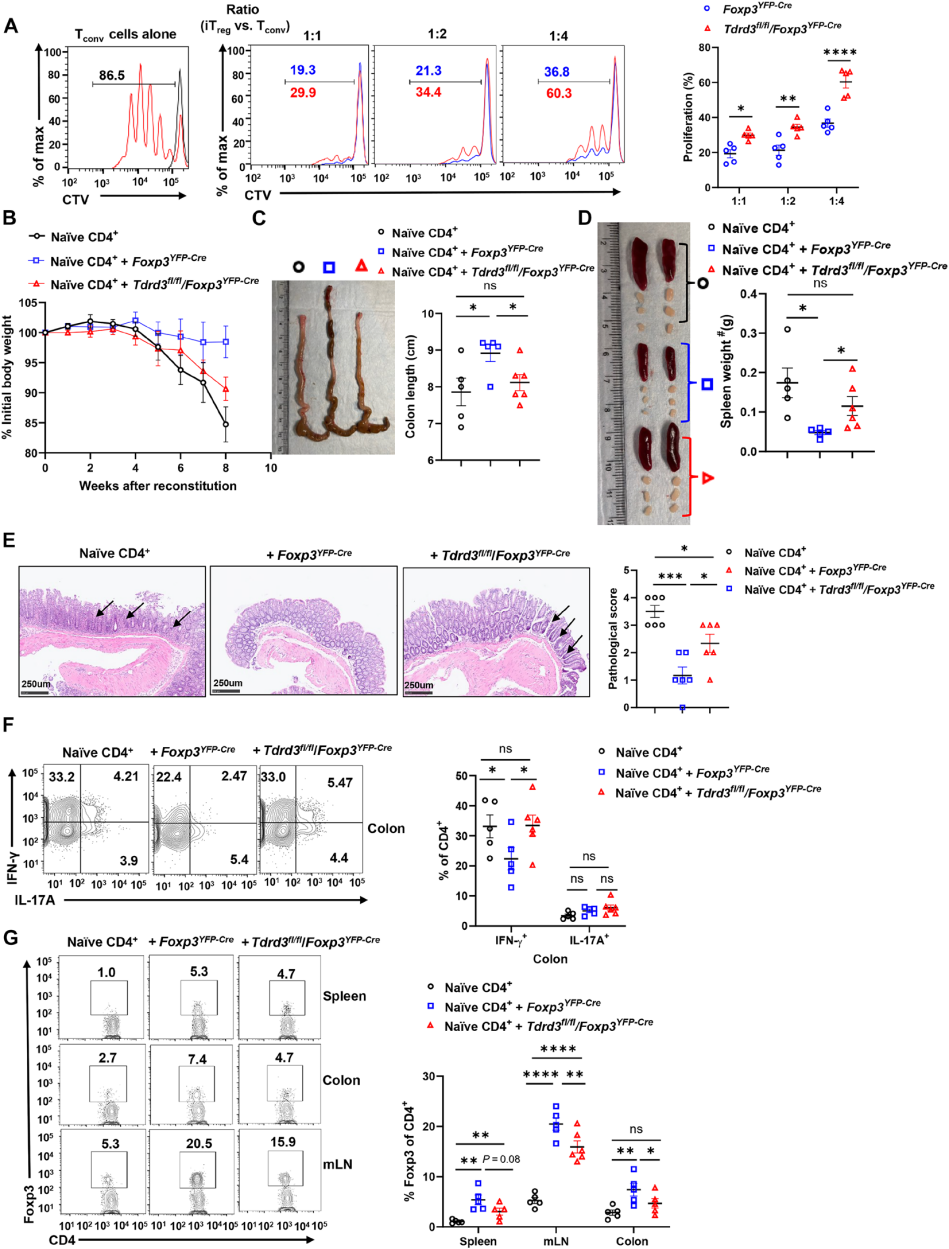
TDRD3 is required for the suppressive function of iT_regs_ but not thymic T_regs_. (**A**) Representative flow cytometric analysis (left panels) and percentage (right) of the proliferating responder CD4^+^ T cells (T_conv_) in the gated area shown on left, labelled with CellTrace Violet dye and cocultured with different ratios of the YFP^+^CD4^+^ iT_regs_ derived from indicated mice (*n* ≥ 4). (**B**) Body weight of *Rag1^−/−^* recipients over time after adoptive transfer of wild-type (WT) naïve CD45RB^hi^CD25^−^CD4^+^ T cells alone or in combination with YFP^+^CD4^+^ iT_regs_ derived from indicated mice. (**C**) Representative image of colons (left) and colon length (right) (*n* ≥ 5) from colitis-induced mice shown in (B). (**D**) Representative image of spleen, lymph nodes (left) and spleen weight (right) (*n* ≥ 5) from colitis-induced mice shown in (B). (**E**) Hematoxylin and eosin (H&E)–stained colon section and their pathological scores from colitis-induced recipients shown in (B), 8 weeks colitis induction. (**F**) Representative flow cytometric analysis (left panels) and percentage (right) of IFNγ^+^ or IL-17A^+^ cells among CD4^+^ T cells in colons of colitis-induced recipients shown in (B) (*n* ≥ 5). (**G**) Representative flow cytometric analysis (left panels) and percentage (right) of Foxp3^+^CD4^+^ T_regs_ among CD4^+^ T cells in spleen, colon, and mLN of colitis-induced recipients shown in (B) (*n* ≥ 5). Boxed area: cell population of interest. Data are from three experiments [(B); (A), (C), (D), (E), (F), and (G), right panels, presented as means ± SEM] or are from one representative of three independent experiments [(A), (C), (D), (E), (F), and (G), left panels]. *P < 0.05, **P < 0.01, ***P < 0.001, and ****P < 0.0005 (two-tailed Student’s *t* test); ns, not significant.

Beyond their differentiation, the ability of regulatory T cells to suppress effector T cell responses is critical for maintaining immune homeostasis. We first assessed the suppressive capacity of in vitro differentiated iT_regs_. iT_regs_ generated from *Tdrd3^fl/fl^/Foxp3^YFP-Cre^* CD4^+^ T cells displayed markedly impaired ability to suppress CD4^+^ T cell proliferation in vitro compared to iT_regs_ derived from *Foxp3^YFP-Cre^* controls (Fig. 3A). We also observed more reduced iT_reg_ derived from *Tdrd3^fl/fl^/Foxp3^YFP-Cre^* CD4^+^ T cells compared to that derived from *Foxp3^YFP-Cre^* controls among corresponding input iT_regs_ at the end of the assay (fig. S3A), indicating likely reduced stability of *Tdrd3^fl/fl^/Foxp3^YFP-Cre^* iT_regs._

We next evaluated the in vivo suppressive function of iT_regs_ using the adoptive transfer colitis model. Transfer of naïve CD4^+^ T cells (CD45RB^hi^CD25^−^CD4^+^) into *Rag1^−/−^* mice induced severe colitis, marked by progressive weight loss (Fig. 3B), shortened colons (Fig. 3C), splenomegaly and enlarged lymph nodes (Fig. 3D), histological evidence of colonic tissue damage (Fig. 3E), and increased frequency of inflammatory IFN-γ–producing (but not IL-17A–producing) CD4^+^ T cells in the colon (Fig. 3F). Cotransfer of iT_regs_ derived from *Foxp3^YFP-Cre^* mice effectively prevented these colitis phenotypes. In contrast, iT_regs_ from *Tdrd3^fl/fl^/Foxp3^YFP-Cre^* mice exhibited impaired ability to confer protection. Consistent with this, the percentage of Foxp3^+^ T_regs_ recovered from spleen, mLNs, and colon was markedly lower in recipients of *Tdrd3*-deficient iT_regs_ compared to those receiving wild-type iT_regs_ (Fig. 3G and fig. S3B for monitoring T_reg_ by YFP). These findings demonstrate that TDRD3 is essential for the suppressive function of iT_regs_.

In contrast, thymic T_regs_ isolated from *Tdrd3^fl/fl^/Foxp3^YFP-Cre^* and *Foxp3^YFP-Cre^* mice were equally effective in suppressing CD4^+^ T cell proliferation in vitro (fig. S3C). Furthermore, cotransfer of thymic T_regs_ from either mouse genotype was equally effective in preventing colitis in vivo, as demonstrated by comparable protective effects across multiple parameters, including body weight changes (fig. S3D), colon length (fig. S3E), spleen and lymph node size (fig. S3F), extent of colon tissue damage (fig. S3G), and IFN-γ production by CD4^+^ T cells in the colon (fig. S3H). Comparable numbers of Foxp3^+^ T_regs_ were recovered from the spleen, colon, and mLN, regardless of genotype of transferred iT_regs_ (fig. S3I). Together, these findings demonstrate that TDRD3 is essential for the suppressive function of iT_regs_ but is dispensable for the inhibitory activity of thymic T_regs_.

### Aged *Tdrd3^fl/fl^/Foxp3^YFP-Cre^* mice develop autoinflammation

**Fig. 4.**
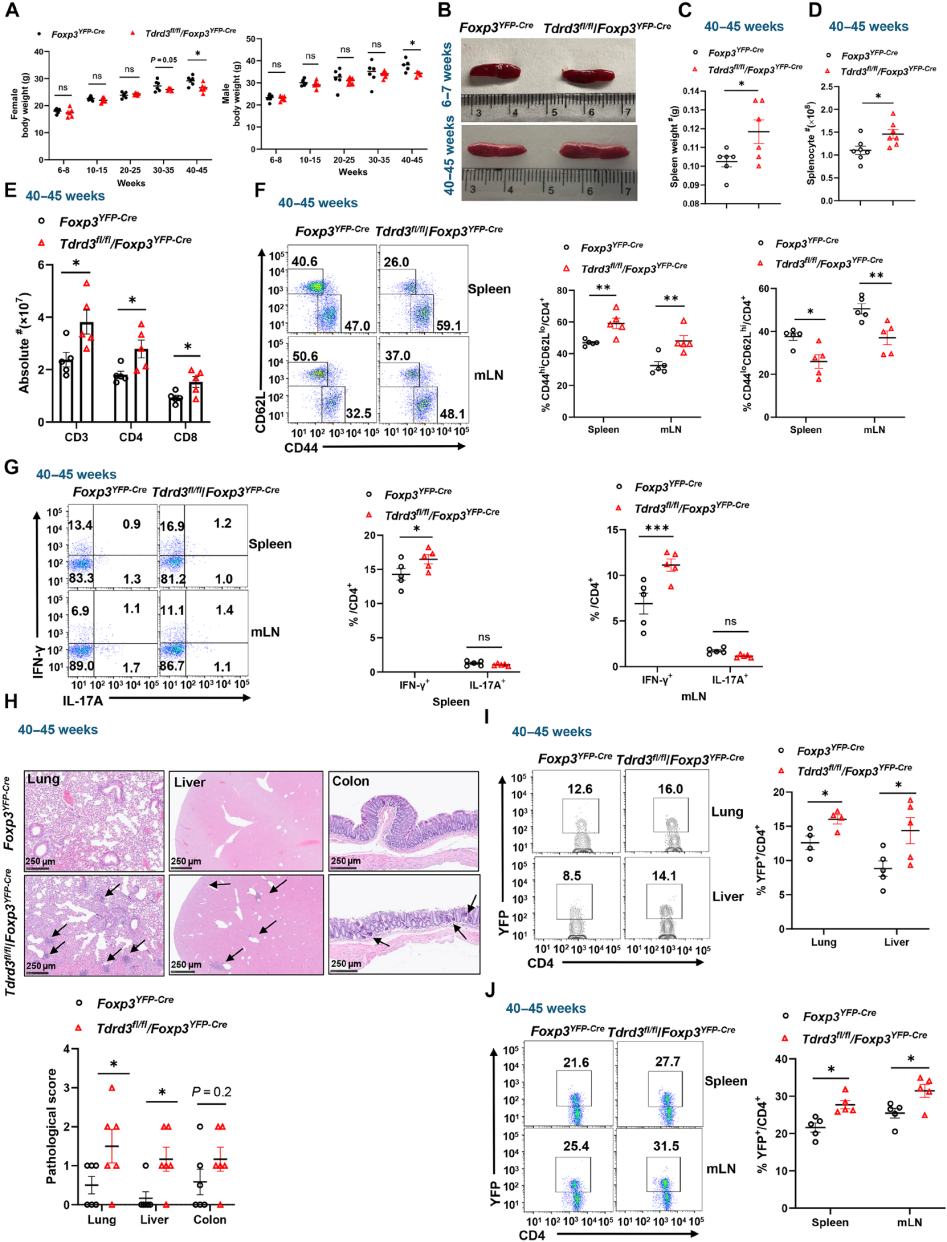
Aged *Tdrd3^fl/fl^/Foxp3^YFP-Cre^* mice develop autoinflammation. (**A**) Body weight of indicated male (left) and female (right) mice at different ages (*n* ≥ 5). (**B**) Representative image of spleens from young (top) and old (bottom) mice of indicated genotypes. (**C** and **D**) Weight (C) and cellularity (D) of the spleens from indicated old mice (*n* ≥ 6). (**E**) Total number of CD3^+^, CD4^+^, and CD8^+^ T cells in spleens from indicated old mice (*n* ≥ 5). (**F**) Representative flow cytometric analysis (left panels) and percentage of CD44^hi^CD62^lo^ memory-like (middle) and CD44^lo^CD62^hi^ naïve (left) cells among splenic CD4^+^ T cells from indicated old mice (*n* ≥ 5). (**G**) Representative flow cytometric analysis (left panels) and percentage of IFN-γ^+^ and IL-17A^+^ cells among CD4^+^ cells from spleens (middle) or mLN (right) of indicated old mice (n ≥ 5). (**H**) Section of H&E–stained (top panels) lung, liver, and colon and their inflammation scores (bottom) from indicated old mice. (**I**) Representative flow cytometric analysis (left panels) and percentage (right) of T_regs_ (Foxp3^+^) among CD4^+^ cells recovered from the lung and liver of indicated old mice (*n* ≥ 4). (**J**) Representative flow cytometric analysis (left panels) and percentage (right panel) of T_regs_ (Foxp3^+^) among CD4^+^ cells recovered from spleen and mLN of indicated old mice (*n* ≥ 5). Boxed area: cell population of interest. Data are from three experiments [(A), (C), (D), and (E); (H) (bottom panel) and (F), (G), (I), and (J) (right panels); presented as means ± SEM] or are from one representative of three independent experiments [(B); (H) (top) and (F), (G), (I), and (J) (left panels)].*P < 0.05, **P < 0.01, and ***P < 0.001 (two-tailed Student’s *t* test); ns, not significant.

We observed that aged (40 to 45 weeks old), but not young (6 to 7 weeks old), *Tdrd3^fl/fl^/Foxp3^YFP-Cre^* mice appeared visibly smaller, a phenotype confirmed by markedly reduced body weight in both males and females compared to age-matched *Foxp3^YFP-Cre^* controls (Fig. 4A). In addition to reduced body weight, old but not young *Tdrd3*-deficient T_reg_ mice also exhibited splenomegaly (Fig. 4B), with markedly increased spleen weight (Fig. 4C) and total cellularity (Fig. 4D), together with an increased CD3^+^ T cells, including both CD4^+^ and CD8^+^ subsets (Fig. 4E). In contrast, spleen weight and cellularity were comparable between young *Tdrd3^fl/fl^/Foxp3^YFP-Cre^* and *Foxp3^YFP-Cre^* mice (fig. S4, A and B). Although older *Foxp3^YFP-Cre^* mice tended to have more CD3^+^ T cells compared to their young controls (fig. S4C), consistent with the notion that older mice generally have aging-associated inflammation (*27*). However, the difference in T cell numbers between young and aged *Foxp3^YFP-Cre^* mice was much less pronounced than that observed between aged *Foxp3^YFP-Cre^* and aged *Tdrd3^fl/fl^/Foxp3^YFP-Cre^* mice (Fig. 4E). These observations indicate that loss of TDRD3 in T_regs_ leads to progressive and spontaneous autoinflammation with age. Supporting this, the phenotypic analysis of splenic and lymph node CD4^+^ T cells revealed a marked increase in CD44^hi^CD62L^lo^ memory-like cells and a corresponding decrease in CD44^lo^CD62L^hi^ naïve T cells in aged *Tdrd3*-deficient T_reg_ mice (Fig. 4F). No such shift was observed in younger mice (fig. S4E).

Further, inflammatory IFN-γ–producing CD4^+^ T cells were markedly elevated in spleens and lymph nodes of aged *Tdrd3^fl/fl^/Foxp3^YFP-Cre^* mice, while IL-17A^+^ cells remained unchanged (Fig. 4G). These increases were not seen in young mice (fig. S4F), reinforcing the age-associated autoinflammation.

Histological analysis further revealed lymphocytic infiltration in the lungs, livers, and colon of aged *Tdrd3^fl/fl^/Foxp3^YFP-Cre^* mice (Fig. 4H). Consistently, the infiltrated liver tissue harbored a higher proportion of inflammatory IFN-γ^+^CD4^+^ T cells but not IL-17A^+^CD4^+^ cells in aged *Tdrd3^fl/fl^/Foxp3^YFP-Cre^* mice (fig. S4G). We also observed reduced inhibitory RORγt^+^Foxp3^+^ cells in colon of aged *Tdrd3^fl/fl^/Foxp3^YFP-Cre^* mice (fig. S4H), consistent with the observed colon inflammation. Intriguingly, although there is no difference in splenic T_regs_ between young *Tdrd3^fl/fl^/Foxp3^YFP-Cre^* and *Foxp3^YFP-Cre^* mice (fig. S4D), the percentage of Foxp3^+^ T_regs_ was increased in the lung, liver (Fig. 4I), spleen, and mLN (Fig. 4J) in old *Tdrd3^fl/fl^/Foxp3^YFP-Cre^* mice compared to *Foxp3^YFP-Cre^* mice. This likely reflects a compensatory expansion of T_regs_ in an attempt to restrain inflammation observed in old *Tdrd3^fl/fl^/Foxp3^YFP-Cre^* mice, a phenomenon previously reported in other mice with similar defective T_regs_ (*28*, *29*). Collectively, these results demonstrate that *Tdrd3* deficiency in T_regs_ leads to a breakdown in immune tolerance over time, culminating in systemic and spontaneous autoinflammation in aged mice.

### TDRD3-mediated up-regulation of *Klf2* is critical for iT_reg_ differentiation and function

**Fig. 5.**
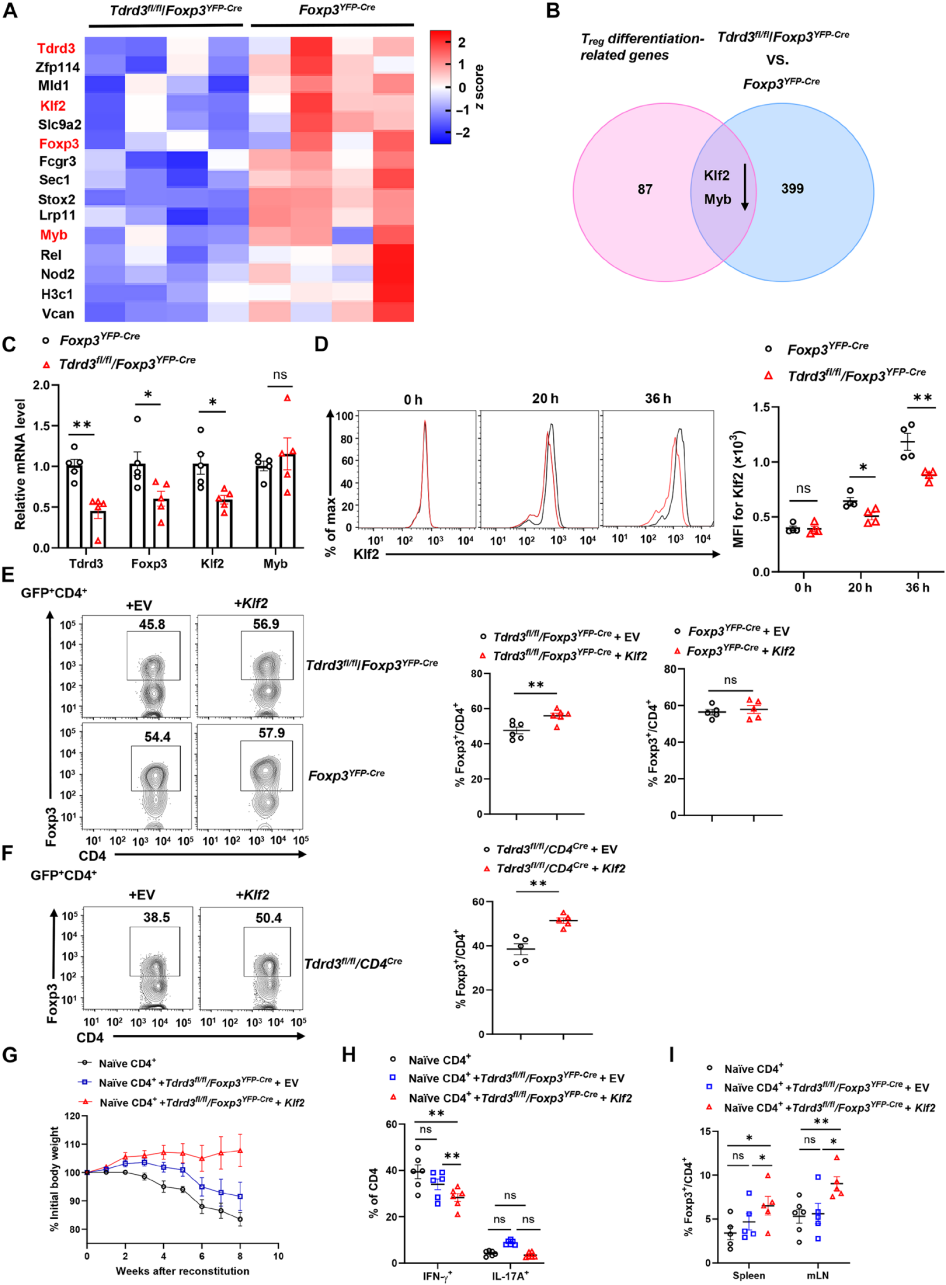
TDRD3-mediated up-regulation of *Klf2* is critical for iT_reg_ differentiation and function. (**A**) Heatmap of genes critical for T_reg_ differentiation from RNA-seq analysis. (**B**) Venn diagram of genes overlapping between 87 iT_reg_ differentiation-related genes and 399 differentially expressed genes (DEGs) between indicated iT_reg_ with a cutoff at *P* < 0.05 and fold change |FC| ≥ 1.2. (**C**) qPCR analysis of *Tdrd3*, *Foxp3*, *Klf2*, and *Myb* mRNAs in indicated iT_reg_ cells (*n* ≥ 3). (**D**) Representative flow cytometric analysis (left panels) of protein and mean fluorescent intensity (MFI; right) for Klf2 in indicated CD4^+^ cells polarized under T_reg_ conditions for 0, 20, or 36 hours (*n* = 4). h, hours. (**E** and **F**) Representative flow cytometric analysis (left panels) and the percentage (right) of Foxp3^+^ T_regs_ among indicated CD4^+^ cells from *Foxp3^YFP-Cre^* and *Tdrd3^fl/fl^/Foxp3^YFP-Cre^* mice (E) or *Tdrd3^fl/fl^*/CD4^Cre^ mice (F), transduced with retrovirus expressing GFP alone (EV) or with *Klf2*, and polarized for 48 hours under T_reg_ conditions (*n* ≥ 4). (**G**) Body weight of *Rag1^−/−^* recipients over time after adoptive transfer of WT naïve CD45RB^hi^CD25^−^CD4^+^ T cells alone or in combination with iT_regs_ differentiated from indicated CD4^+^ T cells retrovirally expressing GFP (EV) along or with *Klf2* and polarized under T_reg_ conditions. (**H**) Percentage of CD4^+^IL-17A^+^ and CD4^+^IFN-γ^+^ cells recovered from colons of colitis-induced recipients shown in (G) (*n* ≥ 4). (**I**) Percentage of YFP^+^CD4^+^ T_regs_ recovered from spleen and mLN of colitis-induced recipients shown in (G) (*n* ≥ 4). GFP, marker of transduction; boxed region, cell population of interest. Data are from three independent experiments [(A), (B), (C), (G), (H), and (I); (D) to (F), right panels; presented as means ± SEM] or are from one representative of three independent experiments [(D) to (F), left panels]. *P < 0.05 and **P < 0.01 (two-tailed Student’s *t* test); ns, not significant.

To elucidate the mechanism underlying TDRD3-regulated iT_reg_ differentiation and given that TDRD3 functions as a transcriptional coregulator involved in gene expression control (*18*, *21*), we performed RNA sequencing (RNA-seq) to compare the transcriptomes of iT_regs_ derived from both genotypes (fig. S5A). The hierarchical clustering of differentially expressed genes revealed clear separation within each group (fig. S5B), highlighting both the reproducibility of the data and the transcriptional impact of gene deletion. As expected, *Tdrd3* and *Foxp3* were among the most down-regulated genes in *Tdrd3*-deficient iT_regs_, consistent with impaired iT_reg_ differentiation upon *Tdrd3* deletion (Fig. 5A).

A total of 399 genes were differentially expressed between *Tdrd3^fl/fl^/Foxp3^YFP-Cre^* and *Foxp3^YFP-Cre^* control iT_regs_. To identify functional regulators, we cross-referenced these 399 genes with 87 genes previously implicated in T_reg_ differentiation based on Gene Expression Omnibus (GEO) datasets and published studies (*29*–*31*) and identified two candidates: *Klf2* and *Myb* (Fig. 5, A and B). Both have been reported as positive regulators of T_reg_ development (*25*, *32*). qPCR analysis confirmed that *Klf2*, along with *Tdrd3* and *Foxp3*, but not *Myb*, was markedly down-regulated in *Tdrd3*-deficient iT_regs_ (Fig. 5C). Consistent with a role in promoting iT_reg_ differentiation, Klf2 protein levels increased during iT_reg_ induction (Fig. 5D). However, this up-regulation was markedly impaired in *Tdrd3^fl/fl^/Foxp3^YFP-Cre^* iT_regs_, mirroring reduced *Klf2* mRNA levels. Moreover, CRISPR-Cas9–mediated knockout of *Klf2* in CD4^+^ T cells from Cas9-expressing mice markedly impaired iT_reg_ differentiation (fig. S5C), confirming its essential role.

To determine whether forced *Klf2* expression can rescue iT_reg_ differentiation in the absence of TDRD3, we retrovirally transduced CD4^+^ T cells from *Tdrd3^fl/fl^/Foxp3^YFP-Cre^* or *Foxp3^YFP-Cre^* mice with constructs expressing green fluorescent protein (GFP) alone [empty vector (EV)] or with *Klf2* (fig. S5D). The expression of *Klf2*, but not GFP alone (EV), restored iT_reg_ differentiation in *Tdrd3^fl/fl^/Foxp3^YFP-Cre^* CD4^+^ T cells (Fig. 5E), whereas the overexpression of *Klf2* in *Foxp3^YFP-Cre^* control cells had no additive effect. Similarly, *Klf2* overexpression in *Tdrd3^fl/fl^/CD4^Cre^* CD4^+^ T cells also enhanced iT_reg_ differentiation (Fig. 5F), placing *Klf2* downstream of TDRD3 in this regulatory pathway.

To test whether *Klf2* also rescues the impaired suppressive function of *Tdrd3*-deficient iT_regs_, we transduced *Tdrd3^fl/fl^/Foxp3^YFP-Cre^* CD4^+^ T cells with *Klf2*-expressing retrovirus and differentiated them into iT_regs_. These cells more effectively suppressed activation-induced proliferation of CD4^+^ T cells in vitro compared to control iT_regs_ expressing only GFP (EV) (fig. S5E). Adoptive transfer of *Klf2*, but not EV, expressing *Tdrd3^fl/fl^/Foxp3^YFP-Cre^* iT_regs_ into *Rag1^−/−^* mice prevented colitis induced by naïve CD4^+^ T cells, as evidenced by protection from weight loss (Fig. 5G), preserved colon length (fig. S5F), and reduced frequency of IFN-γ^+^ inflammatory CD4^+^ T cells in the colon, with no obvious changes in IL-17A^+^CD4^+^ cells (Fig. 5H). A higher percentage of YFP^+^ T_regs_ was also recovered from spleen and mLN of these mice (Fig. 5I), consistent with the reduced inflammation.

Together, these results demonstrate that TDRD3 promotes iT_reg_ differentiation and function by upregulating *Klf2*. The forced expression of *Klf2* is sufficient to restore both the differentiation and suppressive capacity of *Tdrd3*-deficient iT_regs_, establishing TDRD3-*Klf2* as a critical axis in the control of inducible T_reg_ biology.

### TDRD3 recruited by FOXO1 stimulates *Klf2* expression

**Fig. 6.**
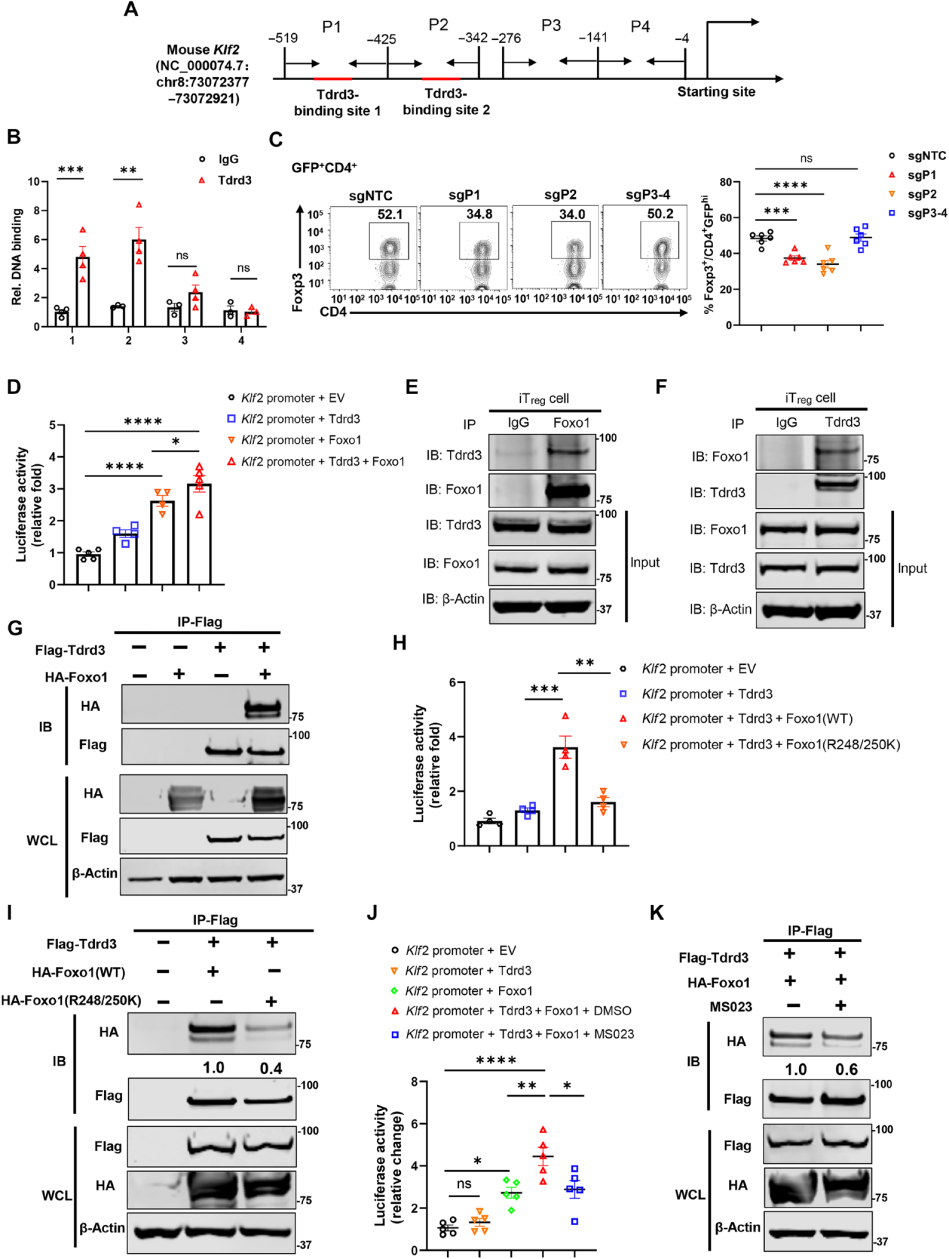
TDRD3 recruited by FOXO1 stimulates *Klf2* expression. (**A**) Sketch of *Klf2* promoter indicating P1 to P4 regions. TDRD3-binding sites (red). (**B**) ChIP analysis of TDRD3 binding to P1 to P4 regions with indicated antibody in iT_regs_ (*n* ≥ 3). (**C**) Cytometric analysis of Foxp3^+^ T_regs_ cells among GFP^+^CD4^+^ cells transduced with nontargeting (sgNTC) or sgP1-P4 targeting guide RNAs and polarized to iT_reg_ (*n* ≥ 3). (**D**) Relative luciferase activity from *Klf2* reporter in human embryonic kidney (HEK) 293T cells with indicated expression plasmids (*n* ≥ 3). (**E** and **F**) Immunoblot (IB) of TDRD3 among FOXO1 or control IgG antibody immunoprecipitation (IP) complexes (E) or Immunoblot of FOXO1 among TDRD3 antibody IP complexes (F) from iT_reg_. Input proteins were detected by immunoblot. (**G**) Immunoblot of HA-FOXO1 among Flag (TDRD3) antibody IP complexes from HEK293T cells with indicated expression plasmids and immunoblot whole cell lysis (WCL) of input proteins. (**H**) Relative luciferase activity from *Klf2* reporter in HEK293T cells with indicated expression plasmids (*n* ≥ 3). (**I**) Immunoblot of HA-FOXO1 among Flag (TDRD3) antibody IP complexes from HEK293T cells with indicated expression plasmids. The number under first row indicates the relative intensity of the band. (WCL) immunoblot of input proteins. (**J**) Relative luciferase activity from *Klf2* reporter in HEK293T cells with indicated expression plasmids ± inhibitor MS023. (*n* ≥ 3). (**K**) Immunoblot of HA-FOXO1 among Flag (TDRD3) IP complexes from HEK293T cells with indicated expression plasmids ± MS203. The number under first row indicates the relative intensity of the band and immunoblot (WCL) of input proteins. Data are from three experiments [(B), (D), (H), and (J); (C), right panels; presented as means ± SEM] or are from one representative of three independent experiments [(E), (F), (G), (I) and (K); (C), left panels]. *P < 0.05, **P < 0.01, ***P < 0.001, and ****P < 0.0005 (two-tailed Student’s *t* test); ns, not significant. HA, hemagglutinin.

To elucidate how TDRD3 regulates *Klf2* expression, we first examined whether TDRD3 binds to the *Klf2* promoter. Based on prior evidence that TDRD3 preferentially associates with promoter regions close to transcription start sites (*18*), we designed primer sets (P1 to P4) to span the proximal 545–base pair (bp) *Klf2* promoter (Fig. 6A). Chromatin immunoprecipitation (ChIP) assays revealed that TDRD3 binds to regions P1 and P2, but not P3 or P4, in iT_regs_ (Fig. 6B).

To test the functional relevance of TDRD3-binding sites, we used CRISPR-Cas9 with paired guide RNAs (gRNAs) to delete individual regions (P1, P2, or P3 to P4) in CD4^+^ T cells from Cas9-expressing mice. Deletions were confirmed by PCR (fig. S6A). Loss of the P1 or P2 regions, but not the P3 to P4 region or a nontargeting control (NTC), markedly impaired iT_reg_ differentiation (Fig. 6C), demonstrating that TDRD3 binding at P1 and P2 is essential for iT_reg_ induction.

Since TDRD3 is a transcriptional coregulator that does not directly bind DNA, we next sought to identify transcription factors that may recruit TDRD3 to the *Klf2* promoter. Based on their known roles in T_reg_ biology and transcriptional regulation, signal transducers and activators of transcription 5 (STAT5) (*33*, *34*), cAMP response element–binding protein (CREB) (*35*–*37*), and FOXO1 (*25*, *38*, *39*) were evaluated as potential TDRD3 partners. Using a luciferase reporter driven by the *Klf2* promoter containing TDRD3-binding sites (Fig. 6A), we found that TDRD3 alone had a minimal effect on reporter activity, whereas FOXO1 activated it (Fig. 6D). The coexpression of TDRD3 and FOXO1 synergistically enhanced *Klf2* promoter activity, while TDRD3 combined with CREB or STAT5 had no such effect (fig. S6B). These results suggest that TDRD3 cooperates specifically with FOXO1 to activate *Klf2* transcription. Further, the single guide RNA (sgRNA)–mediated deletion of *Foxo1* impaired iT_reg_ differentiation (fig. S6C) and decreased Klf2 expression (fig. S6D), supporting positive role of FOXO1 in iT_reg_ differentiation via regulating *Klf2* expression.

To determine whether TDRD3 physically interacts with FOXO1, we performed co-immunoprecipitation assays. TDRD3 was detected in FOXO1 immunoprecipitated complexes from iT_regs_ (Fig. 6E). Conversely, FOXO1 was also found in TDRD3 immunoprecipitated complexes from both iT_regs_ (Fig. 6F) and 293T cells (Fig. 6G), confirming that TDRD3 and FOXO1 are part of the same protein complexes. Notably, this interaction was minimal in naïve CD4^+^ T cells (fig. S6E), suggesting that the TDRD3-FOXO1 interaction is induced upon iT_reg_ polarization and regulates its differentiation.

Given that TDRD3 contains a highly conserved C-terminal tudor domain that recognizes and binds methylated arginine residues (*22*, *23*), we hypothesized that FOXO1 is arginine methylated, which is then recognized by TDRD3, resulting in TDRD3-FOXO1 interaction. FOXO1 has been reported to be methylated at R248 and R250 (*40*). Using an antibody, asymmetric dimethylarginine (ADMA), that recognizes methylarginine (*41*–*43*), we attempted to detect methylation of FOXO1 in both iT_regs_ (fig. S6F, top row of the left panel) and naïve CD4^+^ T cells (fig. S6G, top row of the left panel), but no signal was observed, possibly due to detection limitations of the antibody. We could detect TDRD3 methylation (fig. S6, F and G, top row of the right penal), confirming ADMA in detecting methylarginine.

To functionally test the role of these reported methylation sites, we mutated R248 and R250 to lysine (FOXO1 R248/250 K), which prevents methylation. Unlike wild-type FOXO1, the mutant was unable to cooperate with TDRD3 to activate the *Klf2* promoter in 293T (Fig. 6H) and Jurkat (fig. S6H) cells. In addition, the FOXO1 R248/250K mutant showed reduced interaction with TDRD3 compared to wild-type FOXO1 in immunoprecipitation assays (Fig. 6I), supporting the idea that arginine methylation induces the TDRD3-FOXO1 interaction to stimulate transcription.

Last, we used MS023, a methytransferase inhibitor known to block arginine methylation (*42*, *44*, *45*). MS023 treatment decreased overall ADMA-detected arginine methylation signals (fig. S6I), markedly reduced *Klf2* promoter activity driven by FOXO1 and TDRD3 in both 293T (Fig. 6J) and Jurkat (fig. S6J) cells, and impaired their interaction (Fig. 6K). In addition, the knockdown of endogenous *Foxo1* reduced *Klf2* reporter activity (fig. S6K). Conversely, the coexpression of exogenous FOXO1 with TDRD3 stimulated *Klf2* reporter activity, which was again reduced by Foxo1 knockdown. Additional inhibition of the *Klf2* reporter by MS023 was observed even under *Foxo1* knockdown conditions. This is likely due to incomplete inhibition of *Foxo1* expression by knockdown (fig. S6L). Further, MS023 inhibited iT_reg_ differentiation at day 3 after polarization, while an increase in iT_regs_ was observed at day 2 (fig. S6M). MS023 also impaired the suppressive function of differentiated iT_regs_ (fig. S6N). Although direct detection of FOXO1 methylation by ADMA failed, the functional consequences of the R248/250 K mutation and MS023 treatment support a model in which FOXO1 arginine methylation promotes TDRD3 recruitment, enabling the cooperative activation of *Klf2* expression during iT_reg_ differentiation.

## DISCUSSION

Protein arginine methylation is a well-established posttranslational modification that regulates diverse cellular processes by integrating methylation signals with the transcriptional machinery (*46*, *47*). Dysregulation of arginine methylation has been implicated in a variety of diseases, including inflammatory conditions (*48*, *49*), underscoring its critical role in immune regulation. While numerous studies have investigated the role of protein arginine methyltransferases (PRMTs) in adaptive immune responses—demonstrating their involvement in T cell activation, proliferation, differentiation, and cytokine production—these efforts have largely focused on enzymes such as PRMT1 (*50*, *51*), PRMT5 (*52*–*54*), and PRMT7 (*55*, *56*). In contrast, much less is known about how methylarginine signals are interpreted by their readers, Tudor domain-containing proteins, which bind methylated arginine residues and are believed to mediate downstream transcriptional effects. TDRD3 was first identified as a methylarginine reader (*18*), and its essential roles in transcriptional regulation and genome stability have since been elucidated (*19*–*21*). In this study, we demonstrate that TDRD3 is essential for the differentiation of iT_regs_ from naïve CD4^+^ T cells and for their suppressive function.

T_regs_ originate from two primary sources: differentiation from naïve CD4^+^ T cells in the periphery (iT_regs_) and development in the thymus (thymic T_regs_). Our results show that thymic T_regs_ in *Tdrd3^fl/fl^/Foxp3^YFP-Cre^* mice exhibit normal development and suppressive function. In contrast, iT_reg_ differentiation from naïve CD4^+^ T cells, as well as their suppressive capacity, is impaired in these mice. These findings indicate that TDRD3 is specifically involved in signaling pathways or molecular mechanisms distinctive to iT_reg_ induction and function. Several key differences between iT_regs_ and thymic T_regs_ may underline this differential requirement for TDRD3: (i) Microenvironment and antigen context. iT_reg_ differentiation typically occurs in peripheral tissues under the influence of signals such as TGF-β, retinoic acid, and IL-2, often in response to non–self-antigens such as commensal microbes or food antigens (*57*, *58*). In contrast, thymic T_reg_ development occurs in the thymus during selection against high-affinity self-antigens and is shaped by distinct thymic microenvironmental cues. (ii) Transcription factor requirements. Certain transcription factors, such as members of the nuclear factor of activated T cells (NFAT) family—particularly NFAT1, NFAT2, and NFAT4—are critical for iT_reg_ differentiation but are dispensable for thymic T_reg_ development (*59*). (iii) Foxp3 regulation. The *Foxp3* gene is controlled by multiple conserved noncoding sequences. Conserved noncoding sequence 1, in particular, is required for iT_reg_ differentiation but not for thymic T_reg_ development (*57*). (iv) Surface molecule function. Molecules such as programmed cell death ligand 1 (PD-L1) have been shown to be essential for iT_reg_ induction but are not required for thymic T_reg_ development (*60*). TDRD3 may modulate one or more of these iT_reg_-specific pathways, which could explain its selective importance in iT_reg_ generation and function. Together, our findings suggest that targeting TDRD3 could provide a strategy to manipulate iT_reg_ responses and thereby modulate peripheral immune tolerance.

Both in colitis model and aged *Tdrd3^fl/fl^/Foxp3^YFP-Cre^* mice, we observed apparent up-regulation of IFN-γ–producing T_H_1 cells but not IL-17A–producing T_H_17 cells. This suggests that the deletion of *Tdrd3* in T_regs_ differentially affects T_H_1 and T_H_17 subsets, although both are inflammatory T cells. Several mechanisms may explain this observation. First, T_H_1 and T_H_17 cells are driven by distinct cytokine environments: T_H_1 differentiation critically depends on IL-12 and IFN-γ, whereas T_H_17 differentiation requires TGF-β, IL-6, IL-21, and IL-23 (*61*, *62*). Second, the transcriptional programs governing these lineages are different: T-bet together with STAT1 and STAT4 drives T_H_1 differentiation, while retinoic acid receptor-related orphan receptor gamma t (RORγt) with STAT3 regulates T_H_17 differentiation (*63*–*68*). It is likely that loss of *Tdrd3* in T_regs_ alters the cytokine milieu, enabling dendritic cells, macrophages, or other cells to preferentially produce T_H_1-skewing cytokines, thereby promoting T_H_1 but not T_H_17 differentiation. Since T_regs_ themselves also produce immunomodulatory cytokines, another possibility is that *Tdrd3*-deficient T_regs_ secrete factors that favor T_H_1 polarization. Additional mechanisms may also contribute. For example, IL-2, which promotes T_H_1 while suppressing T_H_17 differentiation (*69*, *70*), could play a role in the preferential expansion of T_H_1 cells. Similarly, checkpoint molecules expressed by T_regs_, such as cytotoxic T lymphocyte-associated antigen 4 (CTLA-4) and T cell immune receptor with Ig and ITIM domains (TIGIT), are known to influence T_H_1 and/or T_H_17 responses in a context-dependent manner (*71*, *72*). Thus, altered expression of these molecules in the absence of *Tdrd3* may further bias the immune response toward T_H_1. Last, it is well established that T_H_17 cells are not stable and can transdifferentiate into T_H_1-like cells (*73*, *74*). This conversion may also contribute to the observed increase in T_H_1 but not T_H_17 cells in *Tdrd3*-deficient settings.

TDRD3 lacks a DNA-binding domain and is therefore typically recruited to transcriptional complexes through interactions with other transcription factors in a methylation-dependent manner. In this study, we demonstrate that TDRD3 interacts with FOXO1 at the proximal *Klf2* promoter region to stimulate *Klf2* expression. *Klf2* has been shown to control iT_reg_ differentiation and function by directly regulating *Foxp3* expression (*25*). *Foxo1* has been shown to play an important role in T_reg_ differentiation, and mice with T_regs_ specific deletion of *Foxo1* display autoinflammation similar to what we observed in *Tdrd3^fl/fl^/Foxp3^YFP-Cre^* mice (*10*, *39*, *75*, *76*). We detected the presence of FOXO1 and TDRD3 in the same protein complexes in iT_regs_—either by immunoprecipitation of FOXO1 or TDRD3—but observed little to no interaction in naïve CD4^+^ T cells, suggesting that the interaction is specifically induced during iT_reg_ differentiation. Although FOXO1 has been reported to be methylated at arginine R248 and R250 (*40*), we were unable to detect methylated FOXO1 using an antibody against methylarginine (ADMA). This may be due to the limited sensitivity of the ADMA antibody, as posttranslational modifications such as methylation can be transient and rapidly removed, resulting in levels below the detection threshold. Notably, the original study identifying FOXO1 methylation at R248/R250 used immunoprecipitated FOXO1 incubated with purified methyltransferase, along with radioactive labeling (*40*), a highly sensitive approach that likely explains why the ADMA antibody could not detect the modification in our setting. Nonetheless, our data support a methylation-dependent interaction between FOXO1 and TDRD3. The mutation of the two reported arginine residues to prevent methylation, or treatment with a protein arginine methyltransferase inhibitor, disrupted the FOXO1-TDRD3 interaction. This strongly supports a role for arginine methylation of FOXO1 in facilitating its association with TDRD3. Furthermore, a functional analysis revealed that mutation of these two arginine residues or inhibition of arginine methyltransferases impaired the ability of FOXO1 and TDRD3 to activate a *Klf2* promoter-luciferase reporter. These findings collectively support a model in which arginine methylation of FOXO1 promotes its interaction with TDRD3, enabling transcriptional activation of *Klf2*, a critical regulator of iT_reg_ differentiation.

Manipulating the differentiation and function of iT_regs_ offers a promising strategy for therapeutic intervention in immune-mediated diseases. iT_regs_ arise from naïve CD4^+^ T cells in the periphery under tolerogenic conditions and play a crucial role in suppressing inflammation and maintaining peripheral tolerance, particularly in mucosal tissues and in response to environmental antigens (*58*). Enhancing iT_reg_ differentiation has shown therapeutic potential in models of autoimmune diseases such as multiple sclerosis and inflammatory bowel disease, where reinforcing immune tolerance can alleviate tissue-damaging inflammation (*77*, *78*). Conversely, inhibiting iT_reg_ generation or destabilizing their suppressive function may enhance antitumor immunity in cancers where T_regs_ limit effective antitumor immune responses (*79*). Thus, targeting the signaling pathways and transcriptional regulators that govern iT_reg_ development is an attractive approach to reprogram immune responses for therapeutic benefit (*80*, *81*). Our study identifies TDRD3 as an essential regulator of iT_reg_ differentiation and suppressive function, highlighting its potential as a therapeutic target. Notably, a TDRD3 inhibitor has recently been developed (*82*), which could be leveraged to enhance immune activation, potentially offering promising avenues for treating immune deficiency or boosting antitumor responses.

## MATERIALS AND METHODS

### Mice

*C57BL* (*B6*, *000664*), *CD4^Cre^* (*TgCd4^cre^*, 022071), and *Rag1^−/−^* (*Rag1^tm1Mom^*, 002216) and mice were purchased from the Jackson Laboratory. *Tdrd3^fl/fl^* mice were obtained from Y.Y.’s Laboratory (Department of Cancer Genetics and Epigenetics, Beckman Research Institute, City of hope, CA), and *Foxp3^YFP-Cre^* mice were obtained from M. Boldin’s laboratory (Molecular and Cellular Biology, Beckman Research Institute, City of Hope, CA). *Tdrd3^fl/fl^* and *Foxp3^YFP-Cre^* mice were crossed to generate the *Tdrd3^fl/fl^/Foxp3^YFP-Cre^* mouse strain. *OT-II* mice were obtained from S. Ma’s laboratory (Department of Hematology and Hematopoietic Cell Transplantation, City of Hope, CA). All mice were bred into the C57BL/6J background and housed under specific pathogen–free conditions in the Animal Resource Center at the Beckman Research Institute of City of Hope under protocols approved by the Institutional Animal Care and Use Committee (IACUC#07023).

### Antibodies and cytokines

Monoclonal antibodies against mouse CD3 (145-2C11), CD4 (RM4–5), CD8 (53–6.7), CD45 (clone 30-F11), CD28 (37.51), IFN-γ (XMG-1.2), CD62L (MEL-14), CD44 (IM7), CD45RB (C363-16A), IFN-γ (XMG-1.2), and CD25 (PC61) were from BioLegend. The antibody against Foxp3 (FJK-16 s), IL-17A (eBio17B7), and Live/Dead Fixable Near-IR Dead Cell Stain (#L34976) were from Invitrogen. The CellTrace Violet Cell Proliferation Kit (#C34571) was from Thermo Fisher Scienctific. Klf2 (#orb124716) was from Biorbyt. Anti-Foxo1 (#2880) antibody, anti-TDRD3 (D3O2G) antibody, anti-ADMA (#13522) antibody, Rabbit immunoglobulin G (IgG) (#2729) antibody and anti–HA-Tag (#5942) antibody were purchased from Cell Signaling Technology. Monoclonal ANTI-FLAG M2 antibody (#F1804) was from Sigma-Aldrich. Recombinant TGF-β (#130-095-067) was from Miltenyi Biotec.

### Plasmids

The retroviral vector murine stem cell virus (MSCV)–internal ribosomal entry site (IRES)–GFP was a gift from W. S. Pear (University of Pennsylvania). 3×Flag-TDRD3 was a gift from Y.Z. Yang (Department of Cancer Genetics and Epigenetics, Beckman Research Institute, City of hope, CA). The cDNA encoding *Klf2* was cloned into MSCV-IRES-GFP vector. The *Klf2* promoter luciferase vector containing a 545-bp promoter of *Klf* in front of the luciferase coding sequence. HA-Foxo1 (pCMV5) (#12142) and retro-gRNA-eGFP (#116926) were purchased from Addgene. PGL3-Basic and pGL3-SV40-Renilla luciferase vectors were purchased from (Promega). HA-Foxo1(R248/250 K) generated by introducing an Arg (R)-to-Lys (K) mutation at amino acid 248/250 of Foxo1 using site-directed mutagenesis (Agilent Technologies).

### Inhibitor

The type I PRMT inhibitor MS023 (#HY-19615) was purchased from MedChemExpress.

### Flow cytometry

For surface staining, cells were directly stained with antibodies and/or fixable live/dead dye in RoboSep buffer (STEMCELL Technologies) at 4°C for 15 min. For transcription factor staining, cells were fixed in TF Fix/Perm buffer (BD Biosciences) at 4°C for 15 min, washed once with TF Perm/Wash buffer (BD Biosciences), and stained with target markers in the TF Perm/Wash buffer at 4°C for 15 min. For intracellular cytokine analysis, cells were stimulated with GolgiStop (BD Biosciences), phorbol 12-myristate 13-acetate (50 ng/ml; Sigma-Aldrich) and ionomycin (750 ng/ml; Sigma-Aldrich) at 37°C for 3 hours in complete culture medium before staining. Following stimulation, cells were first labeled with surface marker antibodies and then subjected to fixation and permeabilization using Cytofix/Cytoperm buffer (BD Biosciences) for 15 min. After washing, intracellular cytokine staining was carried out in Perm/Wash buffer (BD Biosciences). Surface and intracellular marker expression was assessed on a BD LSRFortessa flow cytometer.

### Isolation of naïve CD4^+^ T cells and in vitro T_reg_ differentiation

Naïve CD4^+^ T cells were purified from mouse spleens by negative selection using the Naïve CD4^+^ T Cell Isolation Kit (Miltenyi Biotec). A total of 3 × 10^5^ cells were seeded per well in 48-well plates precoated with rabbit anti-hamster antibody (0.1 mg/ml) and cultured in RPMI 1640 medium (Corning Inc.) supplemented with 2 mM l-glutamine, 50 μM β-mercaptoethanol, penicillin (100 U/ml), streptomycin (100 μg/ml), and 10% fetal bovine serum (Corning Inc.). For T_reg_ differentiation, the culture medium was further supplemented with hamster anti-CD3 (0.25 μg/ml), hamster anti-CD28 (1 μg/ml), TGF-β (5 ng/ml), anti–IL-4 (2.5 μg/ml), and anti–IFN-γ (2.5 μg/ml) and incubated for up to 48 hours.

### In vivo induction of iT_regs_ by adoptively transferring naïve CD4^+^ cells

Naïve CD4^+^ T cells were purified from spleen of *Foxp3^YFP-Cre^* or *Tdrd3^fl/fl^ /Foxp3^YFP-Cre^* mice (8 to 10 weeks) by negative selection using the Naïve CD4^+^ T Cell Isolation Kit, yielding a purity of ≥99.0%. A total of 4 × 10^5^ purified naïve CD4^+^ cells were administered intraperitoneally injected into sex-matched *Rag1^−/−^* mice. Three weeks following adoptive transfer, lymphocytes from colon and mLN of *Rag1^−/−^*-recipient mice were harvest and subjected to analysis.

### In vivo induction of iT_regs_ by oral tolerance

Naïve CD4^+^ T cells were purified from spleen of *OT-II* or *OT-II/ Tdrd3^fl/fl^ /CD4^Cre^* mice (8 to 10 weeks) by negative selection using the Naïve CD4^+^ T Cell Isolation Kit, yielding a purity of ≥99.0%. A total of 3 × 10^6^ cells were administered intraperitoneally injected to sex-matched *Rag1^−/−^* mice. The recipient mice were given access to drinking water containing grade VI OVA (20 mg/ml; Sigma-Aldrich) and libitum for a period of 10 days. The OVA-containing water was replaced every 2 days. On day 10, lymphocytes were isolated from the small intestine, colon, spleen, and mLN for analysis.

### Induction and assessment of EAE

EAE was induced and evaluated following the manufacturer’s protocol (Hooke Laboratories, Lawrence, MA). In brief, *Foxp3^YFP-Cre^* or *Tdrd3^fl/fl^ /Foxp3^YFP-Cre^* female mice (12 to 13 weeks) were immunized with 200 mg of MOG_35–55_ (Hooke Laboratories) emulsified in complete Freund’s adjuvant. Mice subsequently received intraperitoneal injections of pertussis toxin (80 ng) on days 0 and 1. Disease progression was assessed daily using a 0 to 5 clinical scoring system recommended by Hooke Laboratories: 0, no clinical signs; 1, limp tail; 2, hindlimb weakness; 3, complete hindlimb paralysis; 4, both hindlimb and forelimb paralysis; and 5, moribund state or death. Mice euthanized due to severe paralysis were assigned a score of 5 for the remainder of the study.

### In vivo T_reg_ suppression assay

Colitis was induced in sex-matched *Rag1^−/−^* mice through intraperitoneally transfer of 4 × 10^5^ CD45RB^hi^CD25^−^CD4^+^ naïve T cells purified from the spleens of C57BL mice (8 to 10 weeks). For the iT_reg_ suppression assay, iT_regs_ were first induced in vitro from naïve CD4^+^ cells from *Foxp3^YFP-Cre^* or *Tdrd3^fl/fl^/Foxp3^YFP-Cre^* mice for 3 days, CD4^+^YFP^+^ iT_regs_ were sorted out, 2 × 10^5^ iT_regs_ were mixed with 4 × 10^5^ CD45RB^hi^CD25^−^CD4^+^ naïve T cells from C57BL mice and injected into sex-matched *Rag1^−/−^* mice as above. For the thymic T_reg_ suppression assay in *Tdrd3^fl/fl^ /Foxp3^YFP-Cre^* strain mice, 2 × 10^5^ CD4^+^YFP^+^ T_regs_ sorted from the spleen of *Foxp3^YFP-Cre^* or *Tdrd3^fl/fl^/Foxp3^YFP-Cre^* mice were mixed with 4 × 10^5^ CD45RB^hi^CD25^−^CD4^+^ naïve T cells from C57BL mice and injected into sex-matched *Rag1^−/−^* mice. Recipient mice were weighed immediately after transfer and monitored weekly. At 7 to 8 weeks posttransfer, the colon, spleen, and mLN were harvested for downstream analysis.

### In vitro T_reg_ suppression assay

Purified CD4^+^CD25^−^ T cells were labeled with CellTrace Violet (dilution ratio: 1:4000; C34557, Invitrogen) for used as T_resp_ cells. T_resp_ cells (0.5 × 10^5^ cells per well) premixed with anti-CD3/CD28 Dynabeads (Thermo Fisher Scientific) at 1:1 ratio were cocultured with CD4^+^YFP^+^ T_regs_ sorted from the spleens of *Foxp3^YFP-Cre^* or *Tdrd3^fl/fl^/Foxp3^YFP-Cre^* mice in 96-well round-bottom plates for 3 days. For iT_reg_, naïve CD4^+^ cells were purified and cultured under the T_reg_ differentiation condition for 72 hours, and CD4^+^YFP^+^ iT_regs_ were sorted and cocultured with T_resp_ cells for 3 days. The ratios of T_regs_ to T_resp_ cells were 1:1, 1:2, and 1:4 for T_regs_ sorted from mice and the same ratio for iT_regs_ sorted from in vitro differentiation. The proliferation of T_resp_ cells was assessed by flow cytometry.

### Histology study

Tissues were rinsed, fixed in 4% paraformaldehyde, embedded in paraffin, sectioned, and stained with hematoxylin and eosin.

### RNA-seq and analysis

Naïve CD4^+^ T cells purified from *Foxp3^YFP-Cre^* or *Tdrd3^fl/fl^/Foxp3^YFP-Cre^* mice were cultured in 24-well plates under T_reg_-polarizing conditions with TGF-β (5 ng/ml), anti–IL-4, and anti–IFN-γ for 36 hours. Following differentiation, CD4^+^ cells were harvested for RNA isolation using the RNeasy Mini Kit (QIAGEN). For each group, three biological replicates derived from independent mice were included. RNA quality control, library construction, and sequencing were performed by Novogene. The analysis was performed using Illumina Partek Flow version 12.8.1. In brief, the sequence reads were aligned to the mouse whole genome (GRCm39/mm39) using STAR v.2.7.8a followed by validation of quality through prealignment and postalignment quality assurance/quality control. Aligned reads were further subjected to quantification using the Partek E/M algorithm and filtered counts were subjected to normalization and differentially gene expression using DESeq2 algorithm to compare between knockout versus wild-type samples. The statistically significant differential expressed genes were generated by filtering using fold change larger and less than 1.5 and *P* value less than 0.05 to further analyzed using QIAGEN ingenuity pathway analysis (IPA) and pathway analysis.

### Reverse transcription quantitative real-time PCR

Total RNA was isolated using the RNeasy Mini Kit (QIAGEN) following the manufacturer’s instructions. First-strand cDNA was synthesized with the Tetro cDNA Synthesis Kit (Bioline), and qPCR was performed with PowerUp SYBR Green Master Mix (Applied Biosystems) on a QuantStudio 3 Real-Time PCR System (Thermo Fisher Scientific). Primer sequences are provided in table S1, and their amplification efficiencies were validated under optimized reaction conditions. Gene expression was quantified using the ΔΔCt method with β-actin as the internal control, and all reactions were carried out in triplicate.

### Retroviral transduction

Retroviral vectors were transfected into Platinum-E (Plat-E; Cell Biolabs) packaging cells using the BioT transfection reagent (Bioland Scientific). After 24 hours, the medium was replaced, and virus-containing supernatants were collected at 48 and 72 hours, filtered through a 0.45-μm polyvinylidene difluoride syringe filter (Millipore), and either used immediately for T cell transduction or stored at −80°C. Naïve CD4^+^ T cells were labeled with CellTrace Violet (dilution ratio: 1:4000; Invitrogen, C34557) and stimulated for 20 hours on plates precoated with hamster anti-CD3 (0.25 μg/ml) and hamster anti-CD28 (1 μg/ml) antibodies before transduction. Viral transduction was performed by spin infection (2500*g*, 30°C, 2 hours) in the presence of polybrene (10 μg/ml; Sigma-Aldrich). After centrifugation, cells were incubated at 37°C for 3 hours before replacing the viral supernatant with fresh medium containing cytokines and antibodies for T_reg_ polarization.

### Chromatin immunoprecipitation

ChIP was carried out using the ChIP-IT High Sensitivity Kit (#53040, Active Motif). A total of 2 × 10^7^ naïve CD4^+^ cells from *Foxp3^YFP-Cre^* or *Tdrd3^fl/fl^*/*Foxp3^YFP-Cre^* mice differentiated under T_reg_-polarizing conditions were fixed and sheared following the manufacturer’s protocol. ChIP was performed with 30 μg of chromatin using specific antibodies (rabbit IgG and anti-TDRD3, Cell Signaling Technology) and incubated overnight, followed by precipitation with protein G agarose beads. The recovered DNA was analyzed by qPCR to assess enrichment of target sequences, with results normalized to the IgG control. Primer sequences used for ChIP-qPCR are provided in table S1.

### CRISPR-cas9–mediated genomic DNA deletion

To generate genomic deletions of the *Klf2* and *Foxo1* genes using CRISPR-Cas9, sgRNA sequences targeting their coding regions were obtained from the Addgene library (no. 67988) and cloned into the retro-gRNA-eGFP vector. To delete the Tdrd3-binding region of the mouse *Klf2* promoter, two gRNAs targeting the 5′ and 3′ ends of the region were cloned into the retro-gRNA-eGFP vector. These plasmids were co-transfected to produce retrovirus, which was subsequently used to delete the target region in CD4^+^ cells. The sgRNA sequences and primers used to assess the abundance of *Klf2* promoter deletion fragments in genomic DNA are listed in Table S1.

### Luciferase assay

The –545-bp DNA sequence of *Klf2* promoter was inserted between the promoter and luciferase of the PGL3-basic vector serve as *Klf2* promoter luciferase vector. To measure the promoter activity, 1 × 10^6^ human embryonic kidney 293T cells were seeded in six-well plate and transfected the following day using the BioT transfection reagent (Bioland Scientific, Paramount, CA) with the Klf2 promoter luciferase reporter plasmid (1 μg) together with either an empty vector (600 ng), the Tdrd3 expression vector (400 ng), the Foxo1 expression vector (200 ng), or the Foxo1 (R248/250 K) mutant vector (200 ng). To measure the promoter activity in jurkat cell, 1 × 10^6^ Jurkat cell seeded in a six-well plate and transfected the following day using the *Trans*IT-Jurkat transfection reagent (Mirusbio, Madison, WI) with the Klf2 promoter luciferase vector (1 ug) together with expression plasmid for Tdrd3 (500 ng) and/or Foxo1 (500 ng) or Foxo1 (R248/250 K) (500 ng) vector or empty vector (EV). Rinella luciferase vector (100 ng) was cotransfected to cells in each group for normalizing different transfection efficiencies. The total amount of plasmid DNA was adjusted to the same amount using empty vector. Luciferase activity was measured using the Dual-Luciferase Reporter Assay System (Promega, Madison, WI) on a Synergy HTX Multi-Mode Microplate Reader (Agilent, Santa Clara, CA, USA) according to the manufacturer’s instructions.

### Western blotting and immunoprecipitation

For Western blotting, cells were lysed in Pierce RIPA lysis buffer (PI87788, Thermo Fisher Scientific) on ice for 30 min and spun down at 13,000 rpm for 15 min at 4°C to collect the extract. The 4× NuPAGE LDS Sample Buffer was mixed with cell extract and heated at 70°C for 10 mins. Protein was separated by NuPAGE bis-tris gel and transferred to nitrocellulose membrane (Millipore). Target proteins were detected by sequential immunoblotting with specific primary antibodies and fluorescently labeled secondary antibodies (LI-COR Biosciences), and signal intensity was quantified using the Odyssey Imaging System (LI-COR Biosciences). For immunoprecipitation, cells were lysed in Pierce IP Lysis Buffer (PI87787, Thermo Fisher Scientific) supplemented with phenylmethylsulfonyl fluoride (Sigma-Aldrich) and protease inhibitor cocktail (Sigma-Aldrich) on ice for 30 min and spun down at 13,000 rpm for 15 min at 4°C to collect the extract. Five percent of the cell lysate was saved for pre-immunoprecipitation samples. Cell lysates were incubated overnight with specific antibodies, followed by immunoprecipitation with protein A/G Sepharose beads (Santa Cruz Biotechnology, #sc-2003) for 3 hours at 4°C. Beads were washed twice by phosphate-buffered saline and by lysis buffer for the last wash. Beads were then suspended in 4× NuPAGE LDS sample buffer and heated at 70°C for 10 min. The supernatant containing precipitated proteins was subjected to NuPAGE bis-tris gel and analyzed by immunoblot.

### Statistics and reproducibility

The results were analyzed for statistical significance using an unpaired Student’s *t* test or one-way analysis of variance (ANOVA), as appropriate. Data are presented as means ± SEM. *P* values were calculated with GraphPad Prism, and values less than 0.05 (*P* < 0.05) were considered statistically significant.
