# Supplementary material for: TDRD3, a Tudor domain-containing protein, regulates Klf2-dependent Treg differentiation and function to modulate immune tolerance
Source: Sci Adv. 2026 Jan 23;12(4):eaea3960. doi: 10.1126/sciadv.aea3960 (PMC12829583; doi:10.1126/sciadv.aea3960)
Supplement: Supplementary file 1 — Figs. S1 to S7 Table S1 [file sciadv.aea3960_sm.pdf]

Science Advances
AAAS

Supplementary Materials for

TDRD3, a Tudor domain-containing protein, regulates *Klf2*-dependent T_{reg} differentiation and function to modulate immune tolerance

Yun Shi *et al.*

Corresponding author: Yanzhong Yang, yyang@coh.org; Zuoming Sun, zsun@coh.org

Sci. Adv. **12**, eaea3960 (2026)
DOI: 10.1126/sciadv.aea3960

This PDF file includes:

Figs. S1 to S7
Table S1

Figure. S1. TDRD3 is dispensable for thymic T_{reg} development but plays an essential role in the differentiation of regulatory T cells from naïve $CD4^+$ T cells. **A**) Thymic cellularity of indicated mice, quantified by Cellometer (*n* = 8). **B**) Representative flow cytometric analysis of CD4 and CD8 on thymocytes from indicated mice. Right panel: summary of the percentage of $CD4^-CD8^-$ double negative (DN), $CD4^+CD8^+$ double positive (DP), CD4 single positive (SP) and CD8 single positive (SP) thymocytes shown on left ($n \geq 5$). **C**) Representative flow cytometric analysis (left panels) and percentage (right panels) of YFP^- (top panels) and $Foxp3^-$ (bottom panels) cells among naïve $CD4^+$ cells isolated from spleens of indicated mice (*n* = 4). **D**) Representative flow cytometric analysis of YFP and Foxp3 among $CD4^+$ T cells from *Foxp3*$^{YFP\text{-}Cre}$ mice, polarized under T_{reg} conditions, indicating that $CD4^+YFP^+$ cells (left panel) are also $Foxp3^+$ T_{regs} (right panel). **E**) Representative flow cytometric analysis (left panels) and percentage (right panel) of YFP^+ iT_{reg} differentiated *in vitro* from indicated naïve $CD4^+$ cells in the presence of 5ng/ml TGFβ for 48 hours ($n \geq 5$). Boxed region: cell population of interest. Data are from three independent experiments (A; B, C and E, right panels; presented as mean ± SEM) or are from one representative of three independent experiments (D; B, C and E, left panels). **** P<0.0005; ns, not significant (two-tailed Students' t-test).

Figure. S2. TDRD3 is critical for iT_{reg} differentiation and immune regulation *in vivo*. **A**) Gating strategy for Fig. 2D and Fig. 2F ($n \geq 4$). Boxed region: cell population of interest.

Figure. S3. TDRD3 is required for the suppressive function of iT_{regs} but not thymic T_{regs}. **A**) Representative flow cytometric analysis (left panels) and percentage (right panel) of YFP^+ T_{regs} among input $CD4^+$ iT_{regs} derived from indicated mice at the end of this assay shown in Fig. 3A ($n \geq 4$). **B**) Representative flow cytometric analysis (left panels) and percentage (right panel) of YFP^+ T_{regs} among $CD4^+$ T cells recovered from spleen and mesenteric lymph node (mLN) of colitis-induced recipients shown in Fig. 3G ($n \geq 5$). **C**) Representative flow cytometric analysis (left panels) and the relative proliferation (right panel) of responder $CD4^+$ T (T_{conv}) cells cultured with different ratios of thymic YFP^+CD4^+ T_{regs} isolated from indicated mice ($n \geq 4$). **D**) Body weight of *Rag1*$^{-/-}$ recipients over time after adoptive transfer of WT naïve $CD45RB^{hi}CD25^-CD4^+$ T cells alone or in combination with thymic T_{regs} from *Foxp3*$^{YFP\text{-}Cre}$ *and Tdrd3*$^{fl/fl}$/*Foxp3*$^{YFP\text{-}Cre}$ mice ($n \geq 5$). **E**) Image of colons (left panel) and colon length (right panel) from colitis-induced mice shown in D ($n \geq 5$). **F**) Representative image of spleen and lymph nodes (top panel), and spleen weight (bottom panel) ($n \geq 5$ per genotype) from colitis-induced mice shown in D. **G**) H&E-stained colon section from colitis-induced recipients shown in D, 8 weeks after adoptive transfer. **H**) Representative flow cytometric analysis (left panels) and percentage of $IL\text{-}17A^+$ and $IFN\text{-}\gamma^+$ cells among $CD4^+$ T cells recovered from colons (middle panel) or mLN (right panel) of colitis-induced recipients shown in D ($n \geq 5$). **I**) Representative flow cytometric analysis (left panels) and percentage (right panel) of $Foxp3^+$ T_{regs} among $CD4^+$ T cells recovered from spleen, colon, and mesenteric lymph node (mLN) of colitis-induced recipients shown in D ($n \geq 5$). Boxed region: cell population of interest. Data are from three experiments (D; F bottom panel; A, B, C, E, H and I, right panels; presented as mean ± SEM) or are from one representative of three independent experiments (G; F top panel; A, B, C, E, H and I, left panels). **P<0.01, ***P < 0.001, and **** P<0.0005; ns, not significant (two-tailed Students' t-test).

Figure. S4. Aged $Tdrd3^{fl/fl}/Foxp3^{YFP\text{-}Cre}$ mice develop autoinflammation. A-B) Weight (A) and cellularity (B) of the spleens from indicated 6–7-week young mice ($n \geq 4$). **C**) Total number of $CD3^+$, $CD4^+$, and $CD8^+$ T cells in spleens from indicated young and old mice ($n \geq 5$). **D**) Representative flow cytometric analysis (left panels) and percentage (right panel) of T_{regs} among $CD4^+$ cells recovered from spleen of indicated young mice ($n \geq 6$). **E**) Representative flow cytometric analysis (left panels) and percentage (right panels) of $CD44^{hi}CD62^{lo}$ memory-like and $CD44^{lo}CD62^{hi}$ naive cells among splenic $CD4^+$ T cells from indicated young mice ($n \geq 4$). **F**) Representative flow cytometric analysis (left panels) and percentage (right panel) of IL-$17A^+$ and IFN-γ^+ cells among $CD4^+$ T cells recovered from spleen of indicated young mice ($n \geq 4$). **G**) Representative flow cytometric analysis (left panels) and percentage (right panel) of IL-$17A^+$ and IFN-γ^+ cells among $CD4^+$ T cells recovered from liver of indicated aged mice ($n \geq 4$). **H**) Representative flow cytometric analysis (left panels) and percentage (right panel) of $Foxp3^+ROR\gamma t^+$ cells among $CD4^+$ T cells recovered from colon of indicated aged mice ($n \geq 4$). Boxed region: cell population of interest. Data are from three independent experiments (A, B and C; D, E, F, G and H, right panels; presented as mean ± SEM) or are from one representative of three independent experiments (D, E, F, G and H, left panels). $*P < 0.05$, $**P<0.01$, and $***P < 0.001$; not significant (two-tailed Students' t-test).

Figure. S5. TDRD3-mediated upregulation of *Klf2* is critical for iT_{reg} differentiation and function. A) Sketch for RNA-seq analysis (left) of iT_{regs} (right panels) derived from $Foxp3^{YFP\text{-}Cre}$ or $Tdrd3^{fl/fl}/Foxp3^{YFP\text{-}Cre}$ naïve $CD4^+$ T cells. **B**) Heatmap of the transcriptomes in indicated two groups of iT_{regs} differentiated from $Foxp3^{YFP\text{-}Cre}$ (WT) or $Tdrd3^{fl/fl}/Foxp3^{YFP\text{-}Cre}$ (KO) $CD4^+$ T cells. **C**) Representative flow cytometric analysis (left panels) and the percentage (right panel) of $Foxp3^+CD4^+$ T_{regs} among GFP^+CD4^+ T cells retrovirally transduced with non-targeting control (sgNTC) or sg*Klf2* single guide RNA and polarized for 48 hours under T_{reg} conditions ($n \geq 4$). **D**) Representative flow cytometric analysis and mean fluorescence intensity (MFI) of Klf2 among $Foxp3^{YFP\text{-}Cre}$ GFP^+CD4^+ T cells (left two panels) or $Tdrd3^{fl/fl}/Foxp3^{YFP\text{-}Cre}$ GFP^+CD4^+ T cells (right two panels) retrovirally expressing GFP along (EV, empty vector) or with Klf2 under T_{reg}-polarizing conditions for 48 hours ($n = 4$). **E**) Representative flow cytometric analysis (left panels) and percentage (right panel) of the proliferating responder $CD4^+$ T cells (T_{conv}) co-cultured with YFP^+CD4^+ iT_{regs} differentiated from $Tdrd3^{fl/fl}/Foxp3^{YFP\text{-}Cre}$ $CD4^+$ T cells transduced with retrovirus expressing GFP alone (EV) or with *Klf2* ($n \geq 4$). **F**) Representative image of colons (left panel) and colon length (right panel) ($n \geq 5$) from colitis-induced mice shown in Fig 5.G. Boxed region: cell population of interest. Data are from three independent experiments (A, C, D, E and F, right panels; presented as mean ± SEM) or are from one representative of three independent experiments (B; A, C, D, E and F, left panels). $*P < 0.05$, and $**P < 0.01$; ns, not significant (two-tailed Students' t-test).

Figure. S6. TDRD3 recruited by FOXO1 stimulates *Klf2* expression. A) PCR analysis of P1, P2 and P3-4 regions in DNA isolated from Cas9-expressing $CD4^+$ T cells that were transduced with sgNTC, or sgRNAs to confirm the deletion of the corresponding regions. **B**) Relative luciferase activity from *Klf2* reporter transfected into HEK293T cells together with expression plasmid for TDRD3, STAT5, CREB or control empty plasmid (EV) for 32 hours ($n \geq 3$). **C**) Representative flow cytometric analysis (left panels) and the percentage (right panel) of $Foxp3^+$ T_{regs} among indicated $CD4^+$ cells from mice expressing Cas9 mice, transduced with retrovirus

expressing GFP alone or with nontargeting (sgNTC) or targeting Foxo1 (sgFoxo1) guide RNA and polarized for 48 hours under T_{reg} conditions ($n \geq 4$). **D**) Representative flow cytometric analysis (left panels) of protein and mean fluorescent intensity (MFI, right panel) for Klf2 in indicated $CD4^+$ cells polarized under T_{reg} conditions shown in C. **E**) Immunoblot analysis of TDRD3 among protein complexes immunoprecipitated (IP) by anti-FOXO1 or control IgG antibodies from WT naïve $CD4^+$ cells. Input proteins were immunoblotted with anti-TDRD3 or anti-FOXO1 antibody or β-Actin. **F-G**) Immunoblot analysis of FOXO1 and TDRD3 arginine methylation by ADMA among protein complexes immunoprecipitated with anti-FOXO1 (F and G, left panel) or anti-TDRD3 (F and G, right panel) or control IgG antibodies from *in vitro* differentiated iT_{regs} (F) or Naïve $CD4^+$ T cells (G). Input proteins were immunoblotted with anti-TDRD3 or anti-FOXO1 antibody or β-Actin. **H**) Relative luciferase activity from *Klf2* reporter transfected into Jurkat cells together with expression plasmid for TDRD3 and/or FOXO1 or FOXO1 (R248/250R) or empty vector (EV) for 48 hours ($n \geq 3$). **I**) Protein methylation detected by ADMA in cell lysates from HEK293T cells treated with control DMSO or methyltransferases inhibitor MS023. **J**) Relative luciferase activity from *Klf2* reporter transfected into Jurkat cells together with expression plasmid for empty vector (EV) or TDRD3 and FOXO1 in the presence of control DMSO or MS023 for 48 hours. ($n \geq 3$). **K**) Relative luciferase activity from *Klf2* reporter transfected into HEK293T cells transfected with expression plasmid for empty vector (EV) or TDRD3 and FOXO1 together with small interfering RNA nonspecific control (siNC) or targeting Foxo1 (siFoxo1) in the presence of control DMSO or MS023 for 32 hours. **L**) Immunoblot analysis of FOXO1 and loading control actin levels in HEK293T cells transfected with siNC or siFoxo1 shown in K. **M**) Representative flow cytometric analysis (left panels) and the percentage (right panel) of $Foxp3^+$ T_{regs} among WT $CD4^+$ cells polarized under T_{reg} conditions in the presence of DMSO control or MS023 for two (upper panels) or three (lower panels) days ($n \geq 4$). **N**) Representative flow cytometric analysis (left panels) and the relative proliferation (right panel) of responder $CD4^+$ T (T_{conv}) cells cultured with different ratios of indicated YFP^+CD4^+ iT_{regs} differentiated in the presence of control DMSO or MS023 ($n \geq 4$). Boxed region: cell population of interest. Data are from three independent experiments (B, H, J and K; C, D, M and N, right panels; presented as mean ± SEM) or are from one representative of three independent experiments (A, E, F, G, H, I and L; C, D, M and N, left panels). **P* < 0.05, ***P*<0.01, ****P* < 0.001, and **** *P*<0.0005; not significant (two-tailed Students' t-test).

Figure. S7. Full western blotting images within indicated figures.

Fig. S1

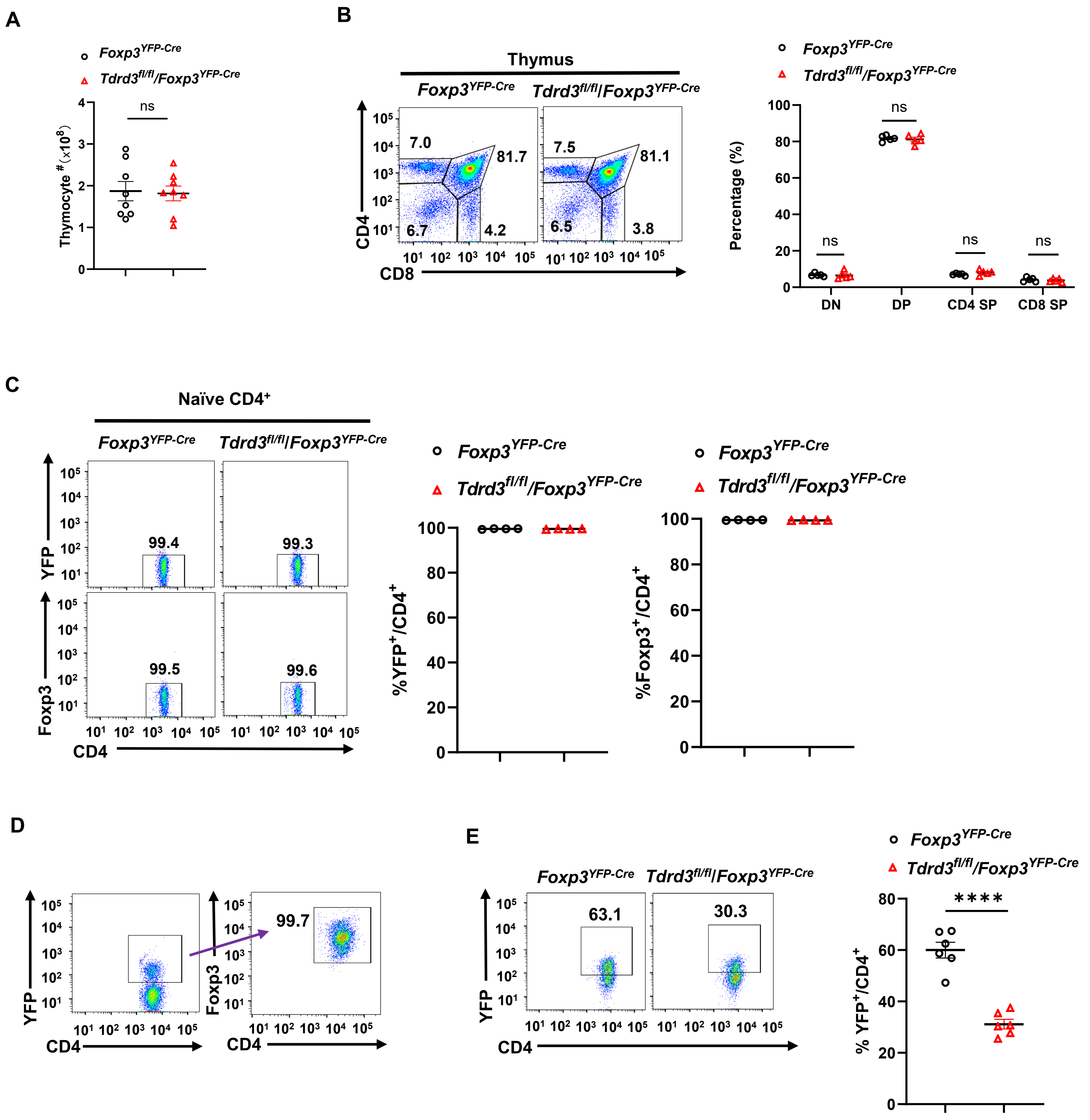
A
Foxp3YFP-Cre
Tdrd3fl/fl/Foxp3YFP-Cre
ns
Thymocyte # (x10⁸)
B
Thymus
Foxp3YFP-Cre
Tdrd3fl/fl/Foxp3YFP-Cre
7.0
81.7
6.7
4.2
7.5
81.1
6.5
3.8
CD4
CD8
Percentage (%)
DN
DP
CD4 SP
CD8 SP
C
Naïve CD4+
99.4
99.3
99.5
99.6
YFP
Foxp3
%YFP+/CD4+
%Foxp3+/CD4+
D
99.7
E
63.1
30.3
****
% YFP+/CD4+

Fig. S2

A

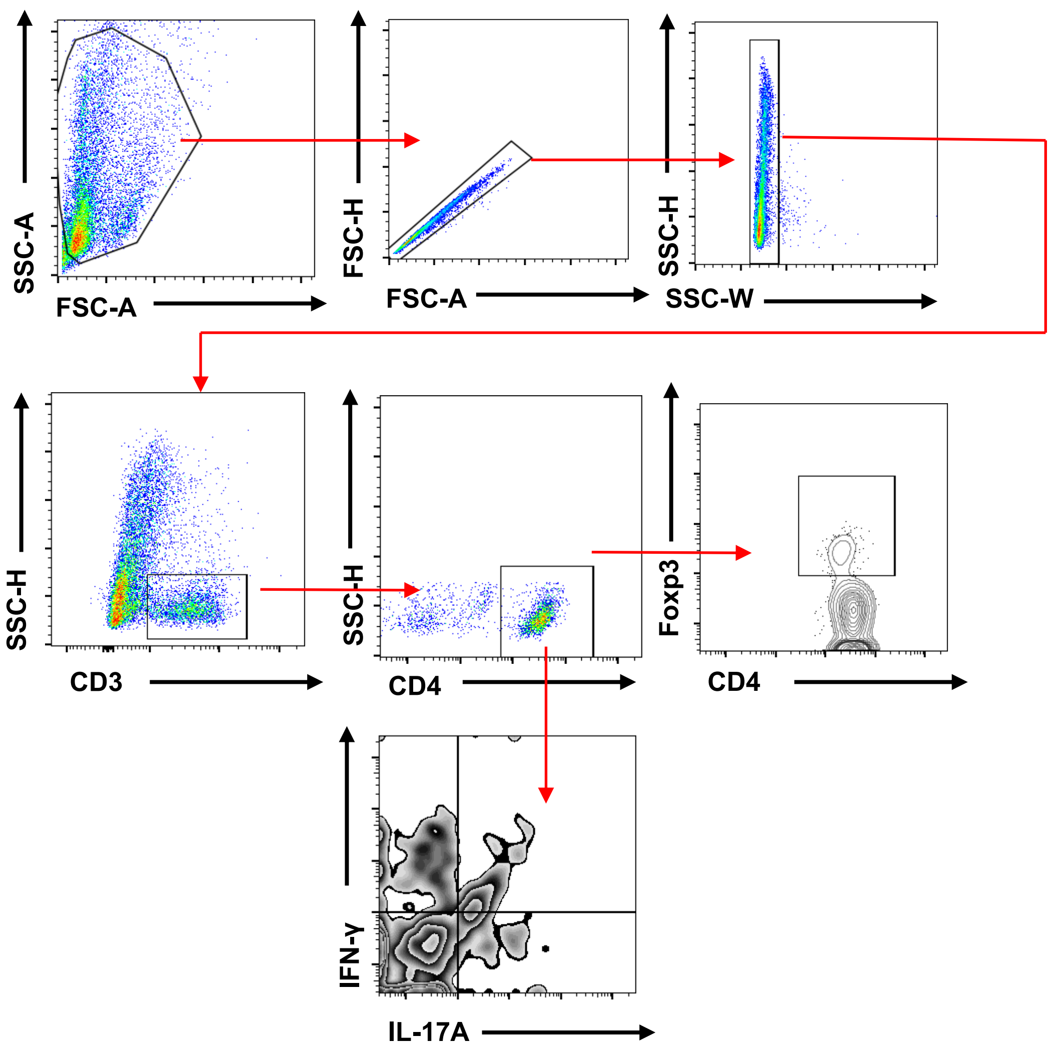
SSC-A
FSC-A
FSC-H
FSC-A
SSC-H
SSC-W
SSC-H
CD3
SSC-H
CD4
Foxp3
CD4
IFN-γ
IL-17A

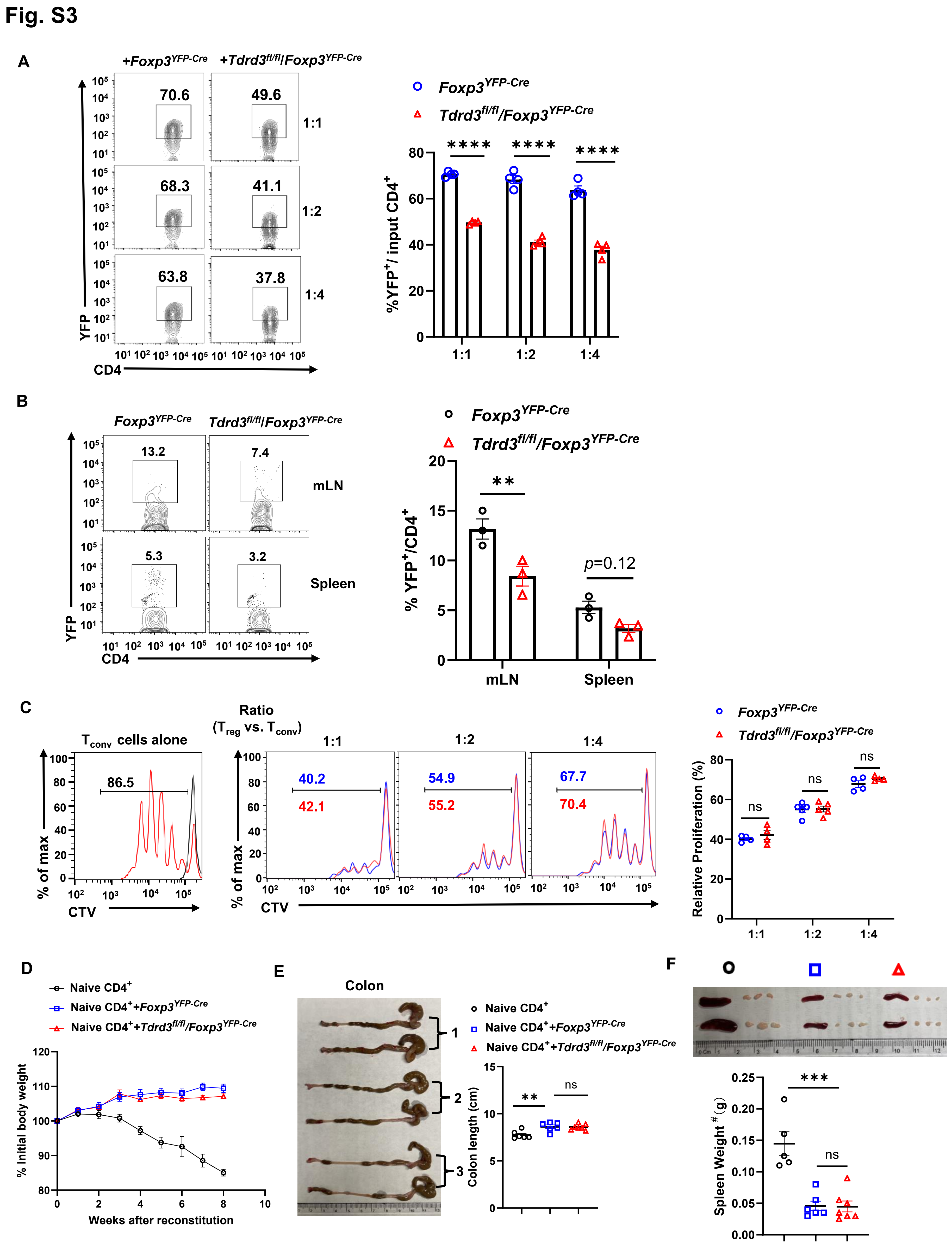
Fig. S3
A
+Foxp3YFP-Cre
+Tdrd3fl/fl/Foxp3YFP-Cre
70.6
49.6
1:1
68.3
41.1
1:2
63.8
37.8
1:4
YFP
CD4
Foxp3YFP-Cre
Tdrd3fl/fl/Foxp3YFP-Cre
%YFP+/ input CD4+
****
1:1
1:2
1:4
B
Foxp3YFP-Cre
Tdrd3fl/fl/Foxp3YFP-Cre
13.2
7.4
mLN
5.3
3.2
Spleen
YFP
CD4
% YFP+/CD4+
**
p=0.12
mLN
Spleen
C
Tconv cells alone
Ratio (Treg vs. Tconv)
86.5
1:1
1:2
1:4
40.2
42.1
54.9
55.2
67.7
70.4
% of max
CTV
Foxp3YFP-Cre
Tdrd3fl/fl/Foxp3YFP-Cre
Relative Proliferation (%)
ns
D
Naive CD4+
Naive CD4++Foxp3YFP-Cre
Naive CD4++Tdrd3fl/fl/Foxp3YFP-Cre
% Initial body weight
Weeks after reconstitution
E
Colon
1
2
3
Naive CD4+
Naive CD4++Foxp3YFP-Cre
Naive CD4++Tdrd3fl/fl/Foxp3YFP-Cre
Colon length (cm)
**
ns
F
Spleen Weight # (g)
***
ns

Fig. S3-continued

G

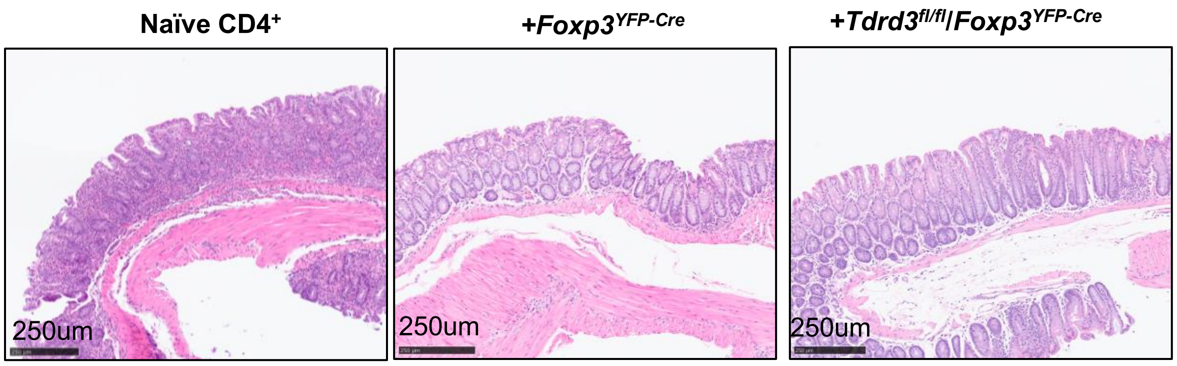

H

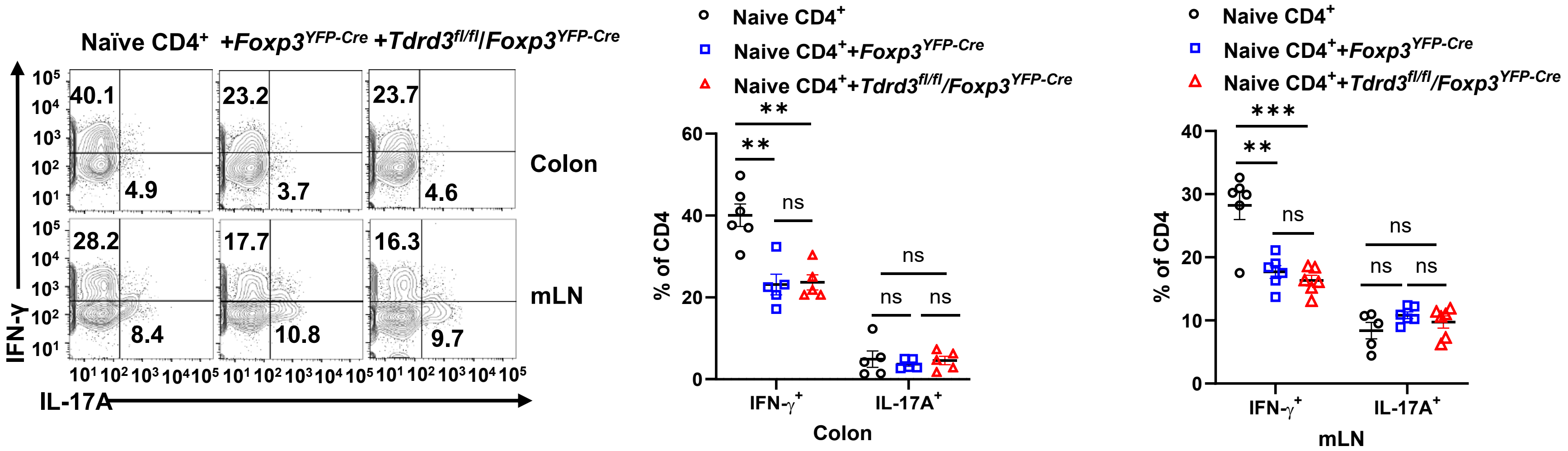

I

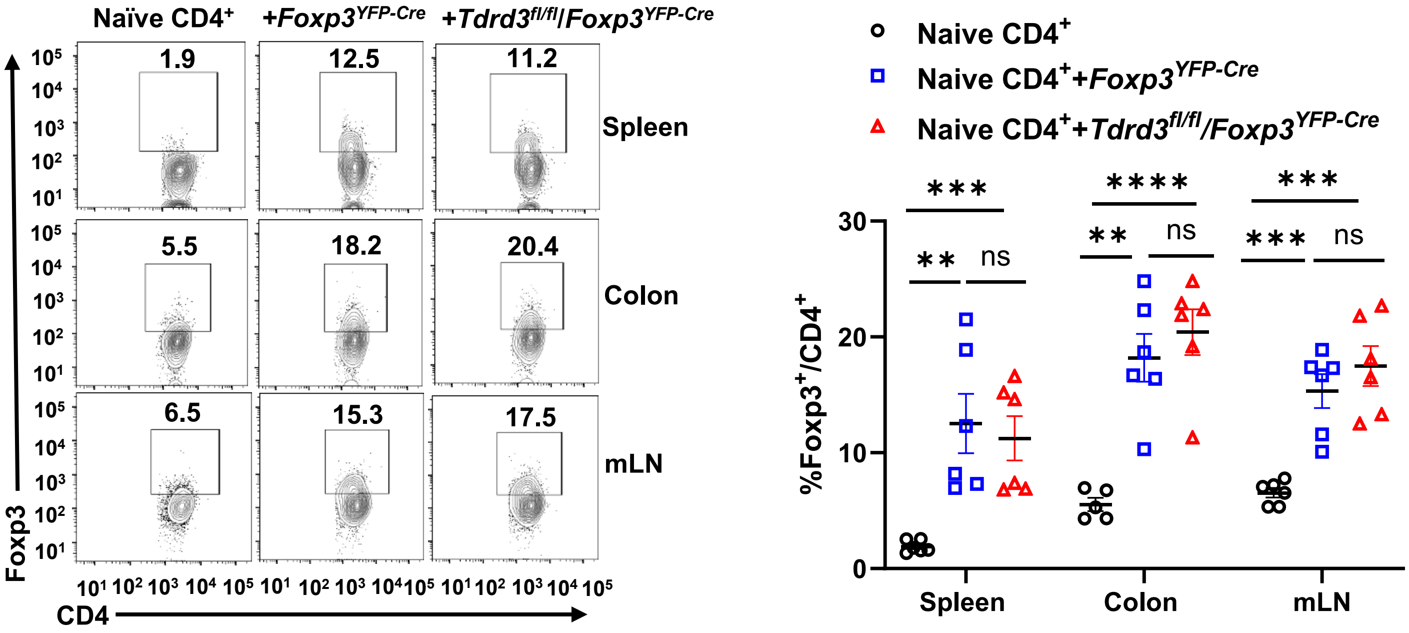

Fig. S4

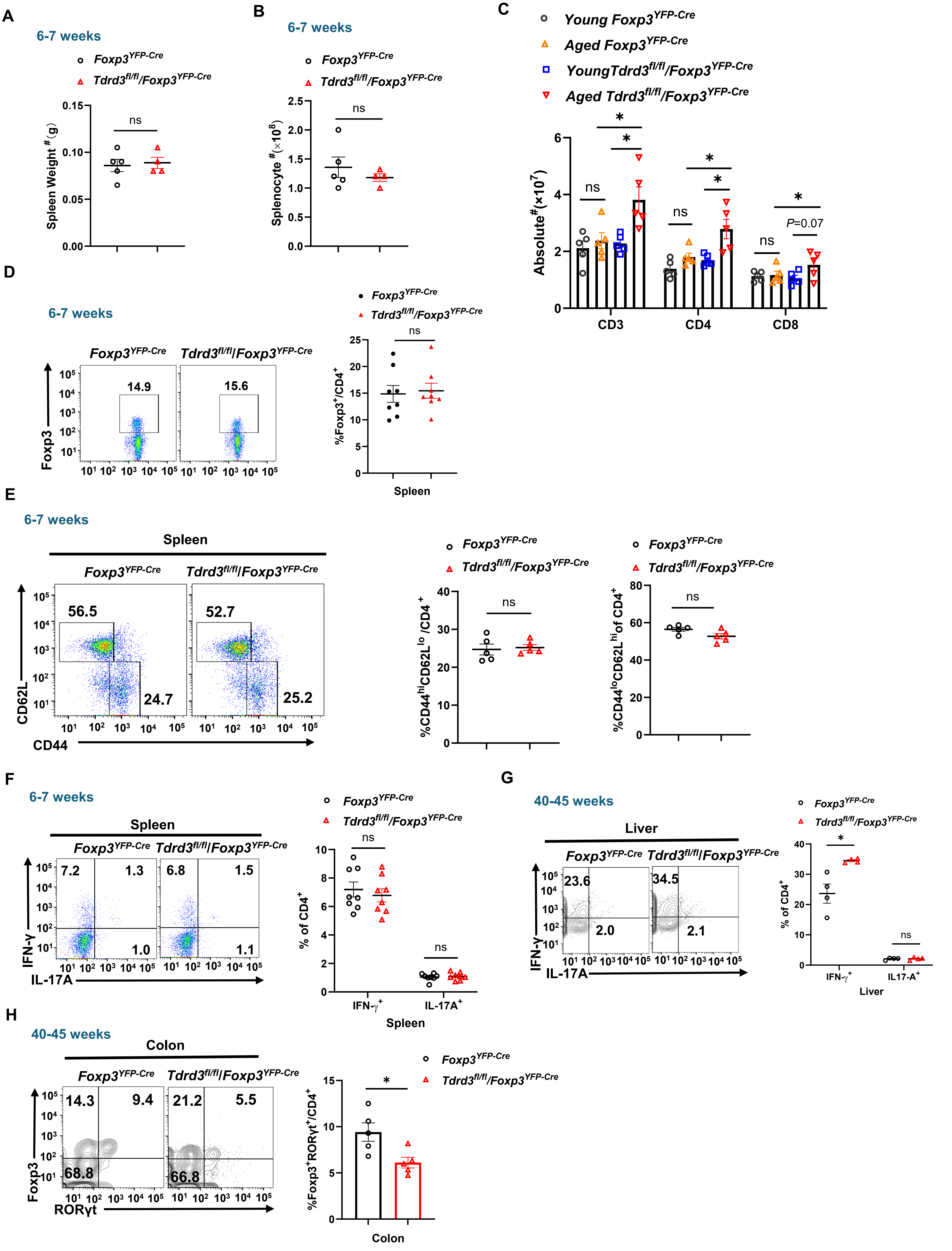

A
6-7 weeks
Foxp3YFP-Cre
Tdrd3fl/fl/Foxp3YFP-Cre
Spleen Weight # (g)
ns
B
6-7 weeks
Splenocyte # (×10^8)
ns
C
Young Foxp3YFP-Cre
Aged Foxp3YFP-Cre
YoungTdrd3fl/fl/Foxp3YFP-Cre
Aged Tdrd3fl/fl/Foxp3YFP-Cre
Absolute # (×10^7)
P=0.07
CD3
CD4
CD8
D
6-7 weeks
Foxp3
14.9
15.6
%Foxp3+/CD4+
Spleen
E
6-7 weeks
Spleen
CD62L
CD44
56.5
24.7
52.7
25.2
%CD44hiCD62Llo/CD4+
%CD44loCD62Lhi of CD4+
F
6-7 weeks
Spleen
IFN-γ
IL-17A
7.2
1.3
1.0
6.8
1.5
1.1
% of CD4+
IFN-γ+
IL-17A+
G
40-45 weeks
Liver
23.6
2.0
34.5
2.1
IL17-A+
H
40-45 weeks
Colon
Foxp3
RORγt
14.3
9.4
68.8
21.2
5.5
66.8
%Foxp3+RORγt+/CD4+

Fig. S5

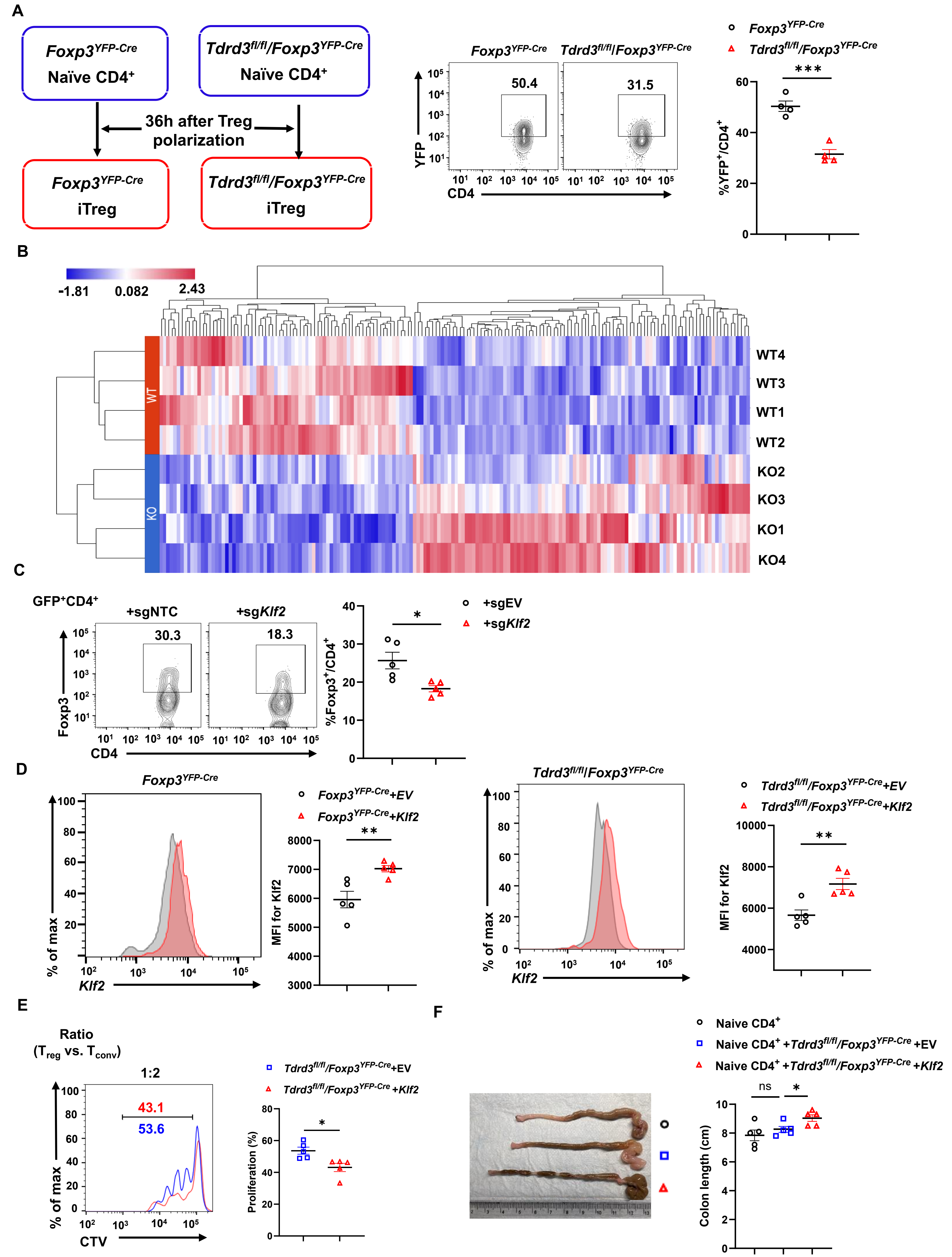
A
Foxp3YFP-Cre Naïve CD4+
Tdrd3fl/fl/Foxp3YFP-Cre Naïve CD4+
36h after Treg polarization
Foxp3YFP-Cre iTreg
Tdrd3fl/fl/Foxp3YFP-Cre iTreg
50.4
31.5
YFP
CD4
%YFP+/CD4+
***
B
-1.81
0.082
2.43
WT
KO
WT4
WT3
WT1
WT2
KO2
KO3
KO1
KO4
C
GFP+CD4+
+sgNTC
+sgKlf2
30.3
18.3
Foxp3
CD4
%Foxp3+/CD4+
+sgEV
+sgKlf2
*
D
Foxp3YFP-Cre
Foxp3YFP-Cre+EV
Foxp3YFP-Cre+Klf2
% of max
Klf2
MFI for Klf2
**
Tdrd3fl/fl/Foxp3YFP-Cre
Tdrd3fl/fl/Foxp3YFP-Cre+EV
Tdrd3fl/fl/Foxp3YFP-Cre+Klf2
E
Ratio
(Treg vs. Tconv)
1:2
43.1
53.6
CTV
Proliferation (%)
F
Naive CD4+
Naive CD4+ +Tdrd3fl/fl/Foxp3YFP-Cre +EV
Naive CD4+ +Tdrd3fl/fl/Foxp3YFP-Cre +Klf2
ns
Colon length (cm)

Fig. S6

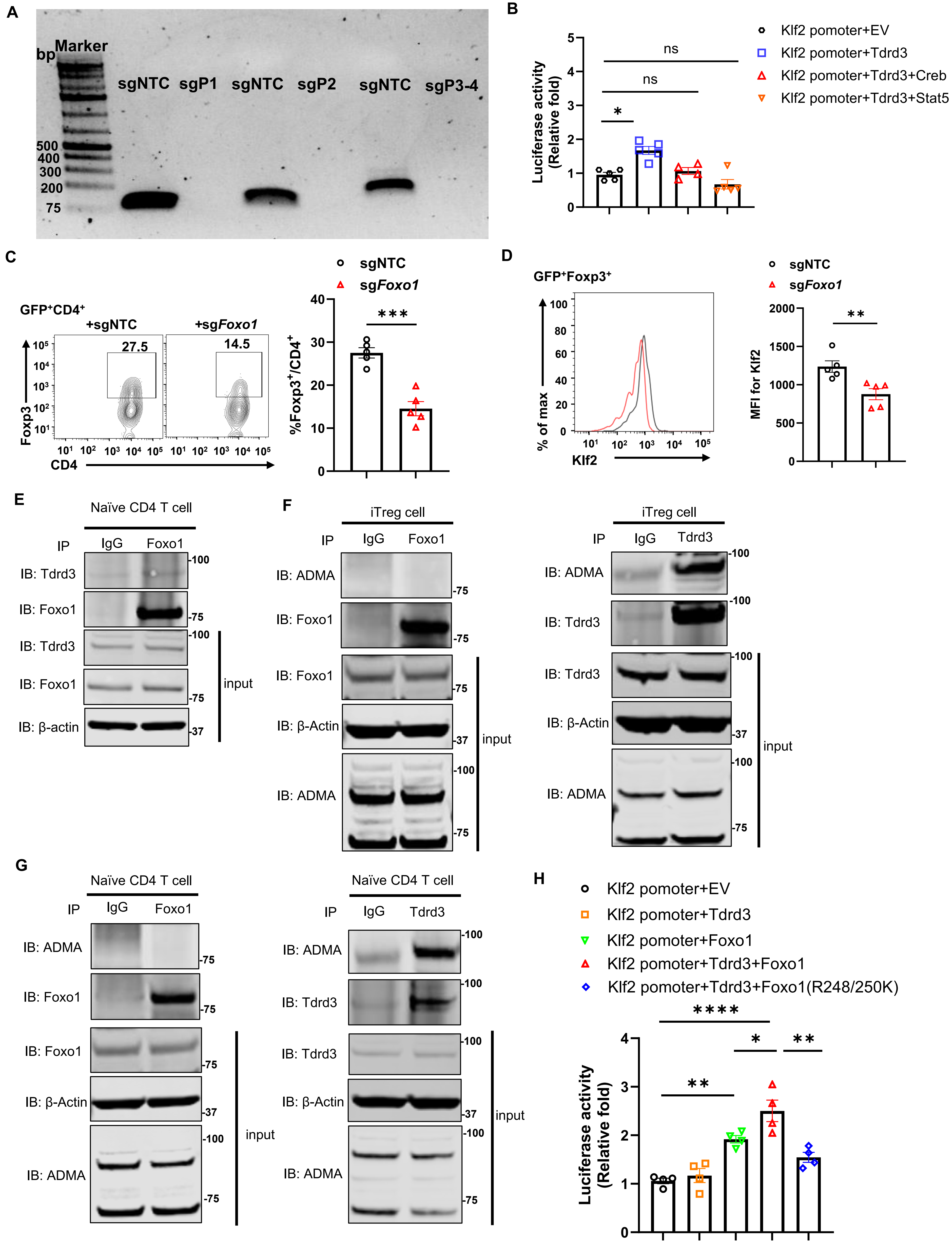
A
bp
Marker
500
400
300
200
75
sgNTC
sgP1
sgNTC
sgP2
sgNTC
sgP3-4
B
Luciferase activity (Relative fold)
Klf2 pomoter+EV
Klf2 pomoter+Tdrd3
Klf2 pomoter+Tdrd3+Creb
Klf2 pomoter+Tdrd3+Stat5
ns
ns
*
C
GFP⁺CD4⁺
+sgNTC
+sgFoxo1
27.5
14.5
Foxp3
CD4
sgNTC
sgFoxo1
%Foxp3⁺/CD4⁺
***
D
GFP⁺Foxp3⁺
% of max
Klf2
sgNTC
sgFoxo1
MFI for Klf2
**
E
Naïve CD4 T cell
IP
IgG
Foxo1
IB: Tdrd3
IB: Foxo1
IB: Tdrd3
IB: Foxo1
IB: β-actin
input
F
iTreg cell
IP
IgG
Foxo1
IB: ADMA
IB: Foxo1
IB: Foxo1
IB: β-Actin
IB: ADMA
input
iTreg cell
IP
IgG
Tdrd3
IB: ADMA
IB: Tdrd3
IB: Tdrd3
IB: β-Actin
IB: ADMA
input
G
Naïve CD4 T cell
IP
IgG
Foxo1
IB: ADMA
IB: Foxo1
IB: Foxo1
IB: β-Actin
IB: ADMA
input
Naïve CD4 T cell
IP
IgG
Tdrd3
IB: ADMA
IB: Tdrd3
IB: Tdrd3
IB: β-Actin
IB: ADMA
input
H
Klf2 pomoter+EV
Klf2 pomoter+Tdrd3
Klf2 pomoter+Foxo1
Klf2 pomoter+Tdrd3+Foxo1
Klf2 pomoter+Tdrd3+Foxo1(R248/250K)
Luciferase activity (Relative fold)
****
*
**
**

Fig. S6-continued

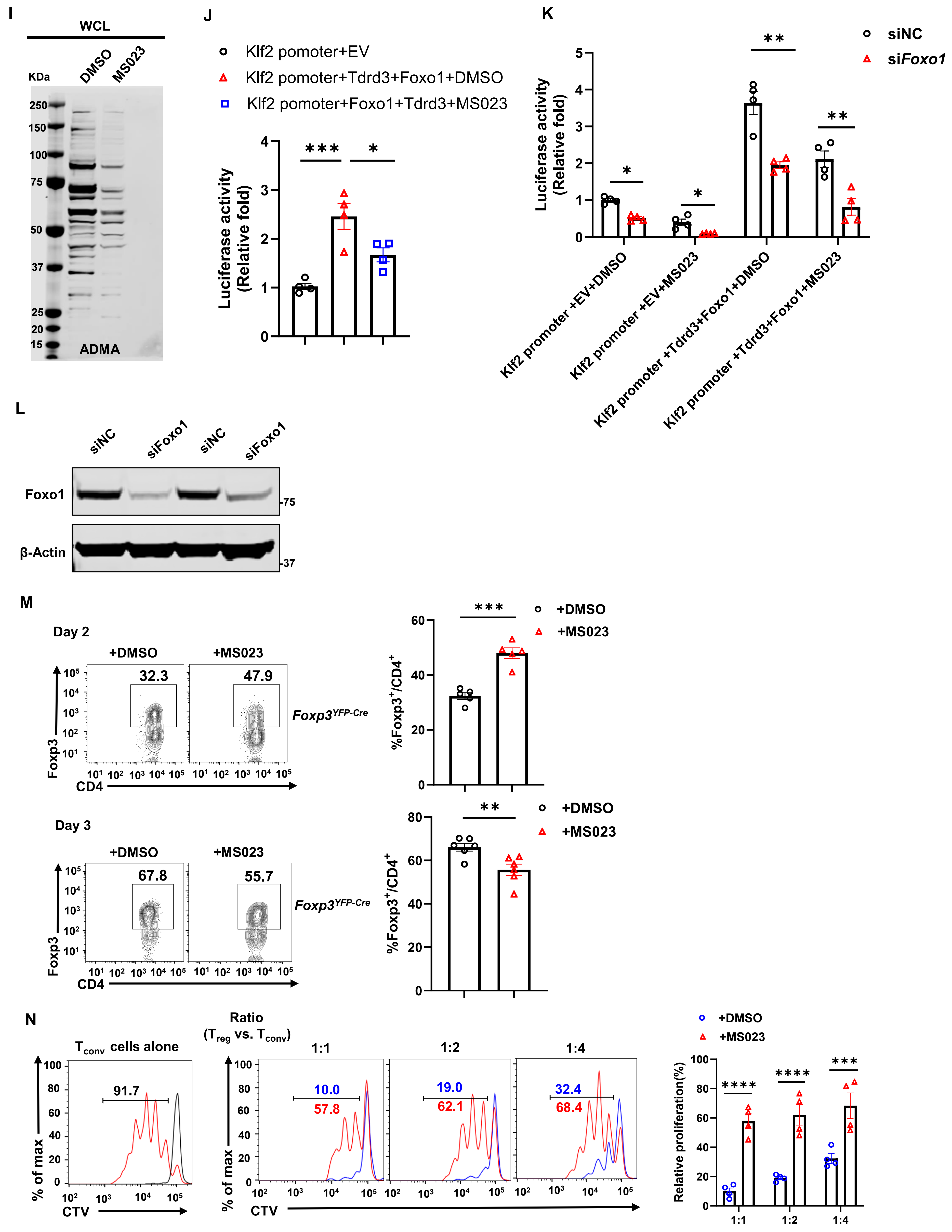

Fig. S7

Fig. 1 A

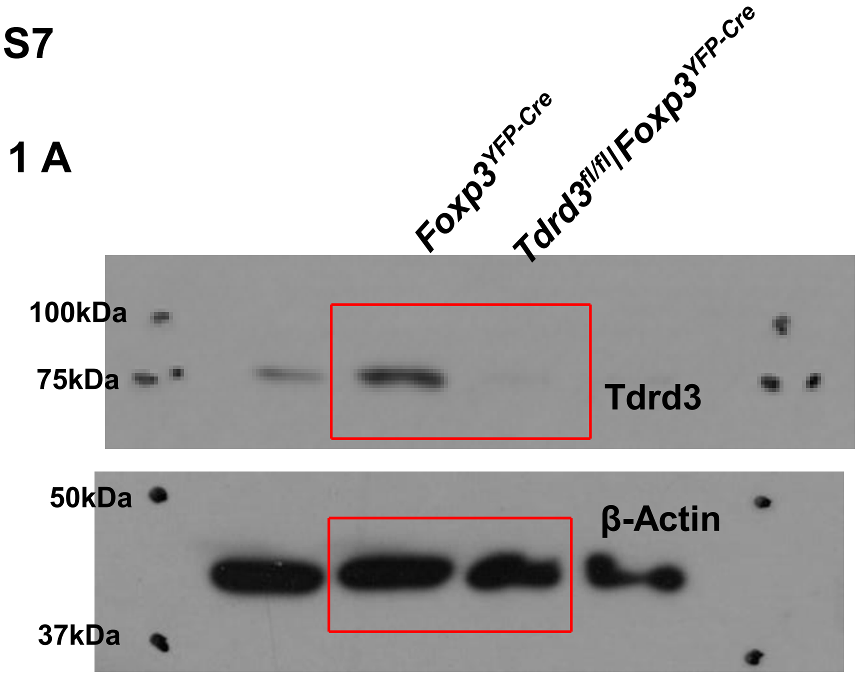

Fig. 1 B

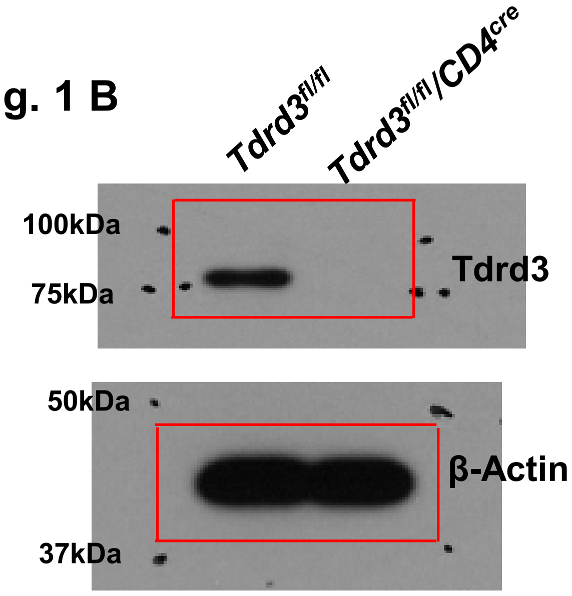

Fig. 6E

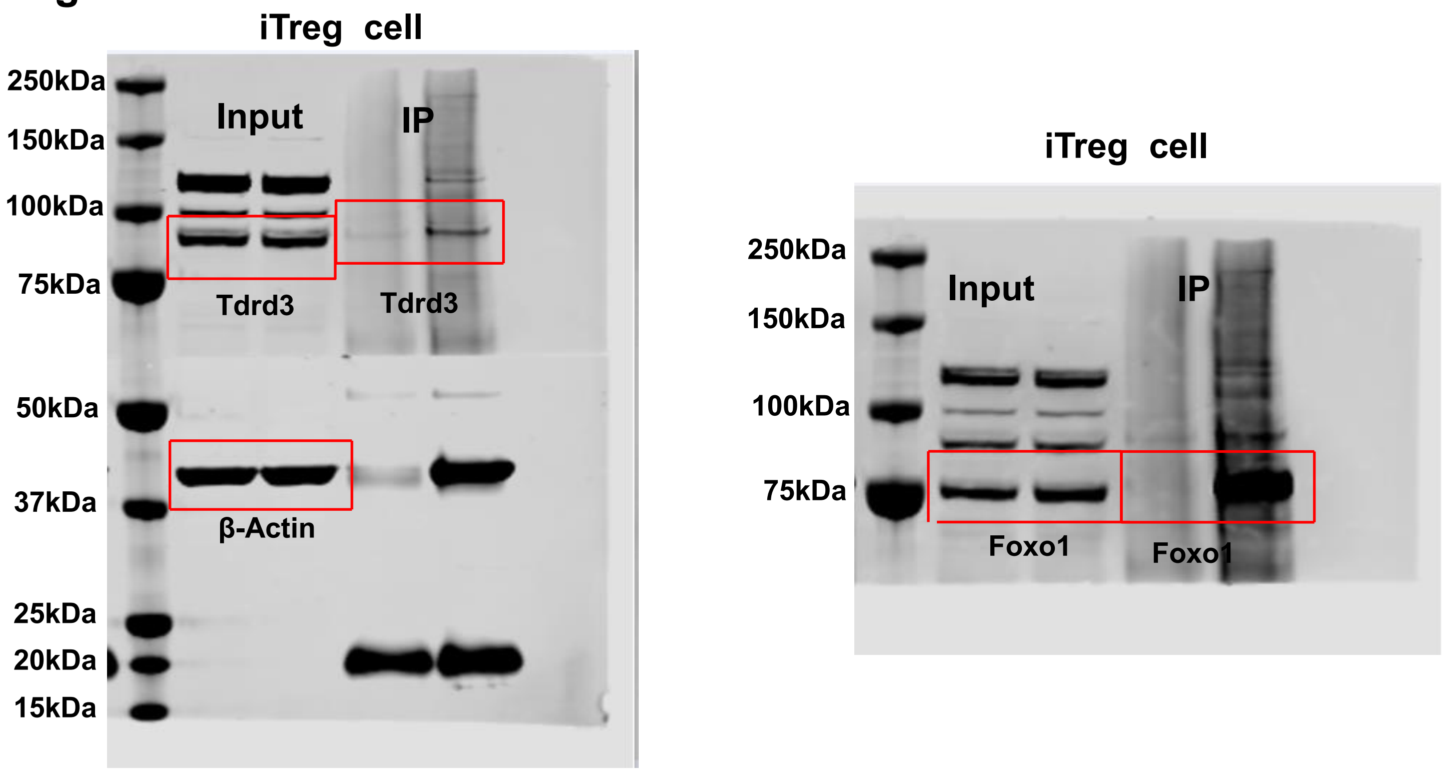

Fig. 6F

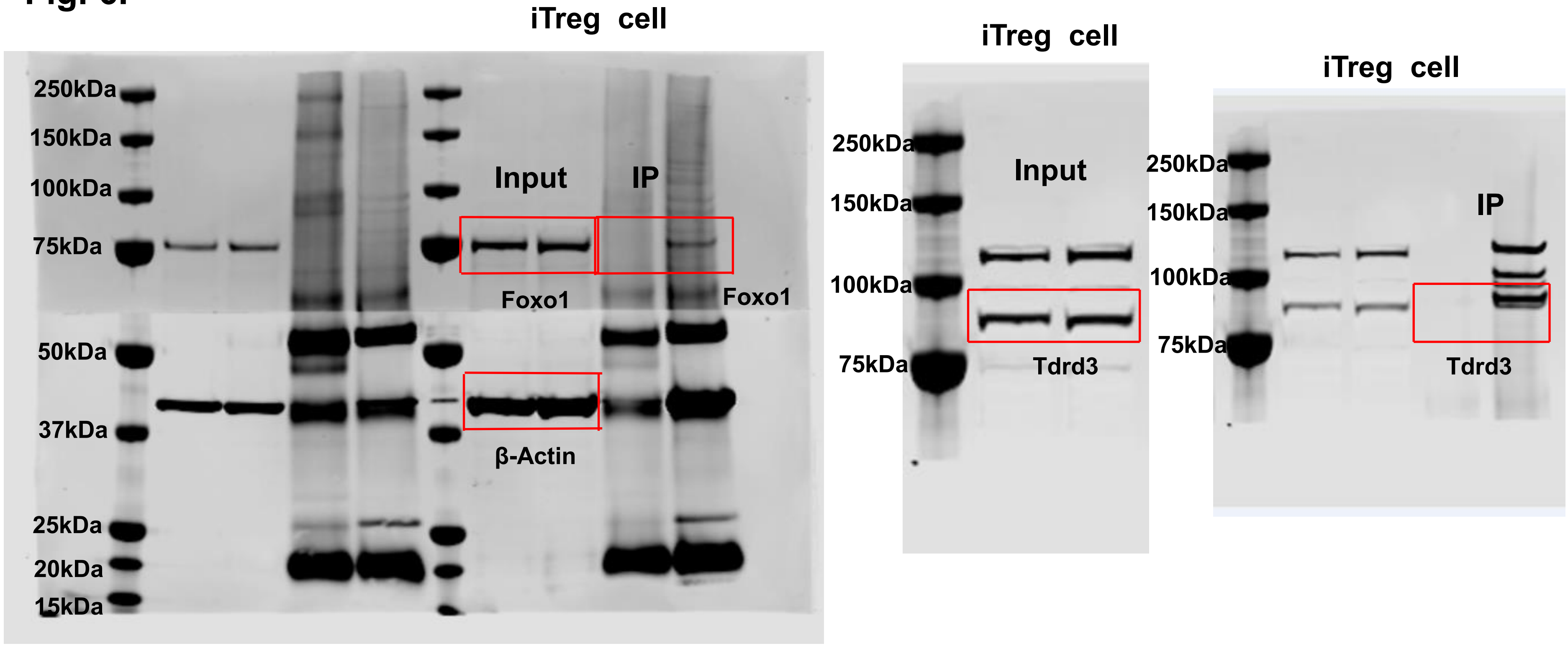

Fig. S7-continued

Fig. 6 G

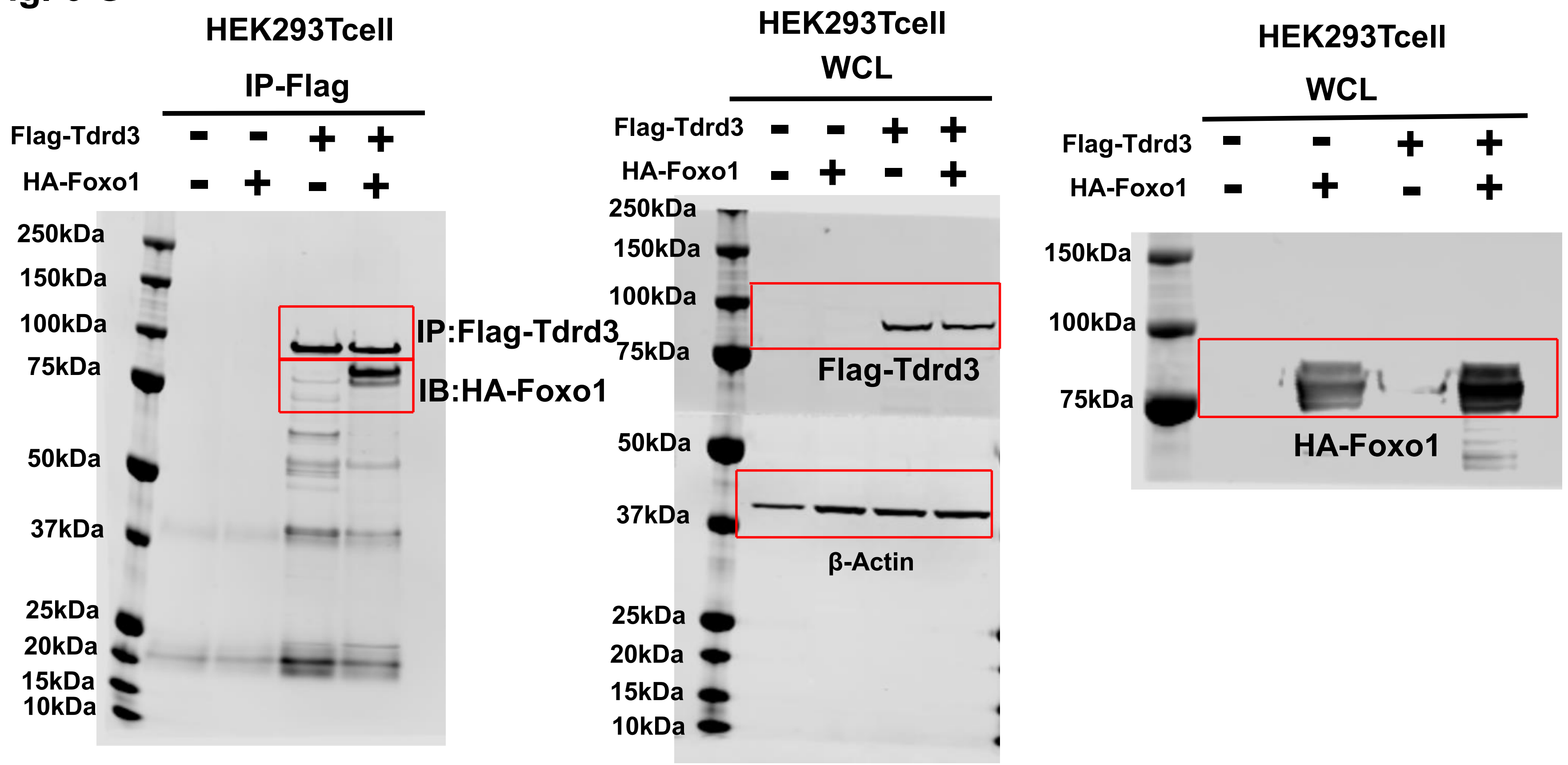

Fig. 6 I

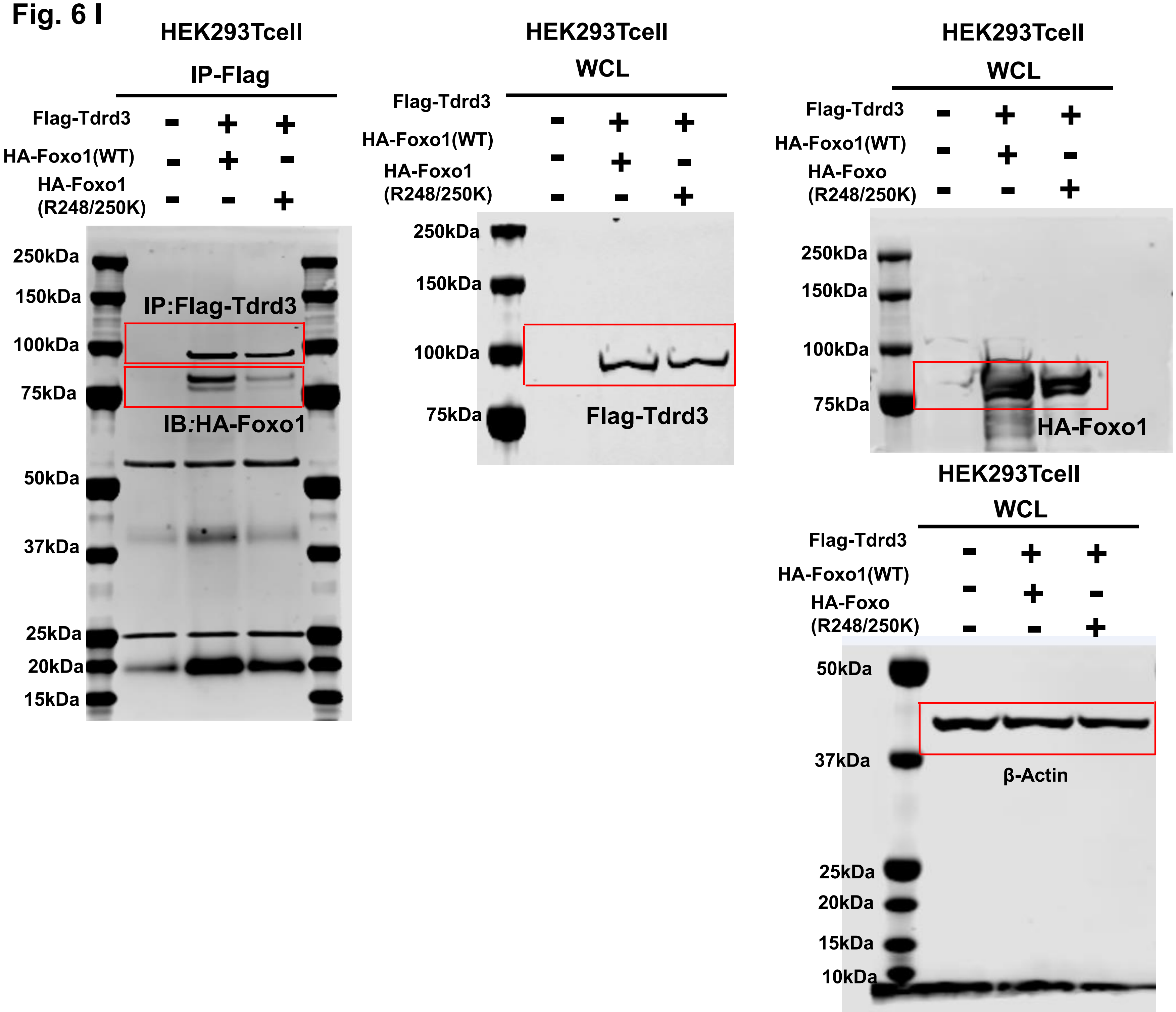

Fig. S7-continued

Fig. 6 K

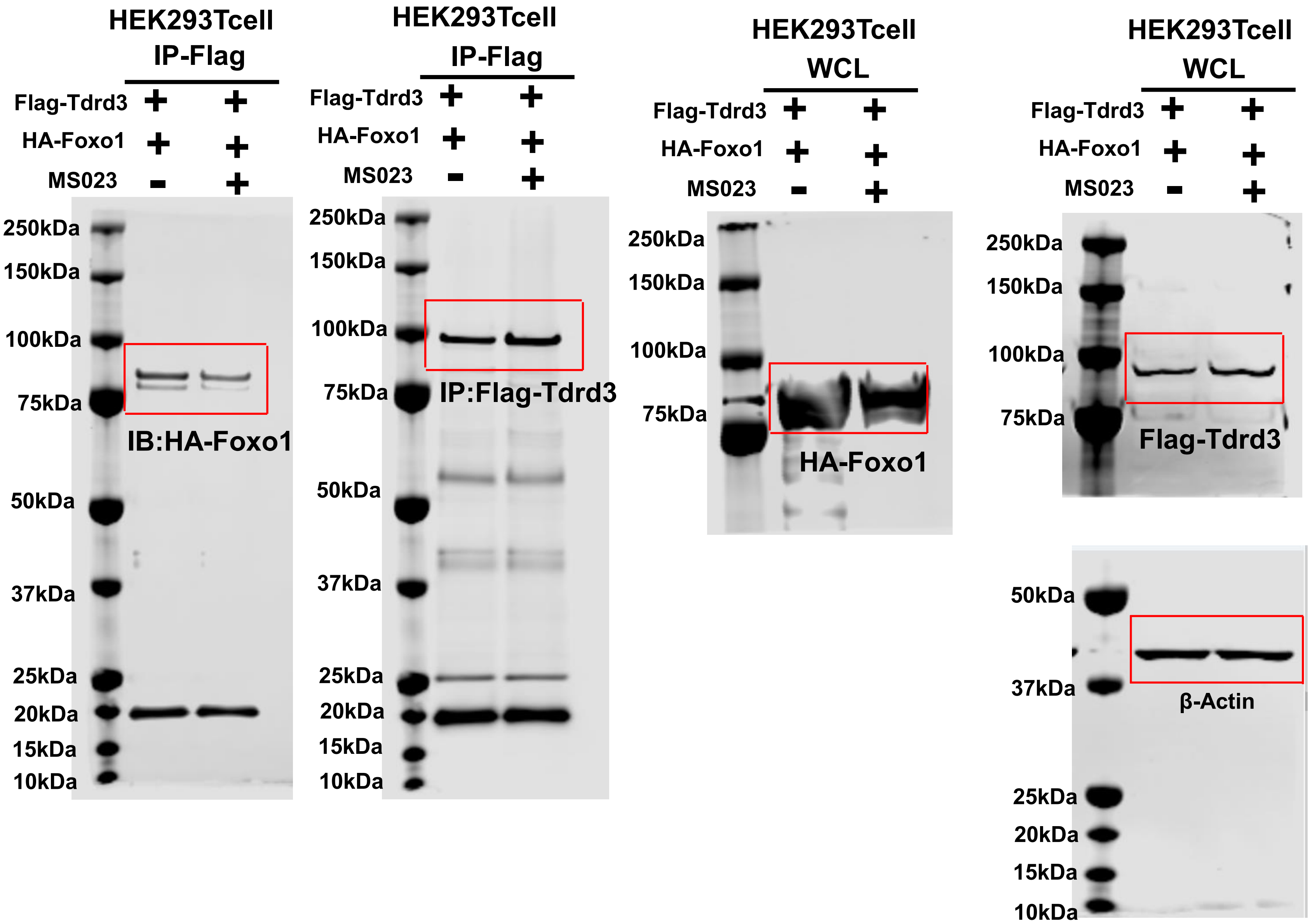

fig. S6 E

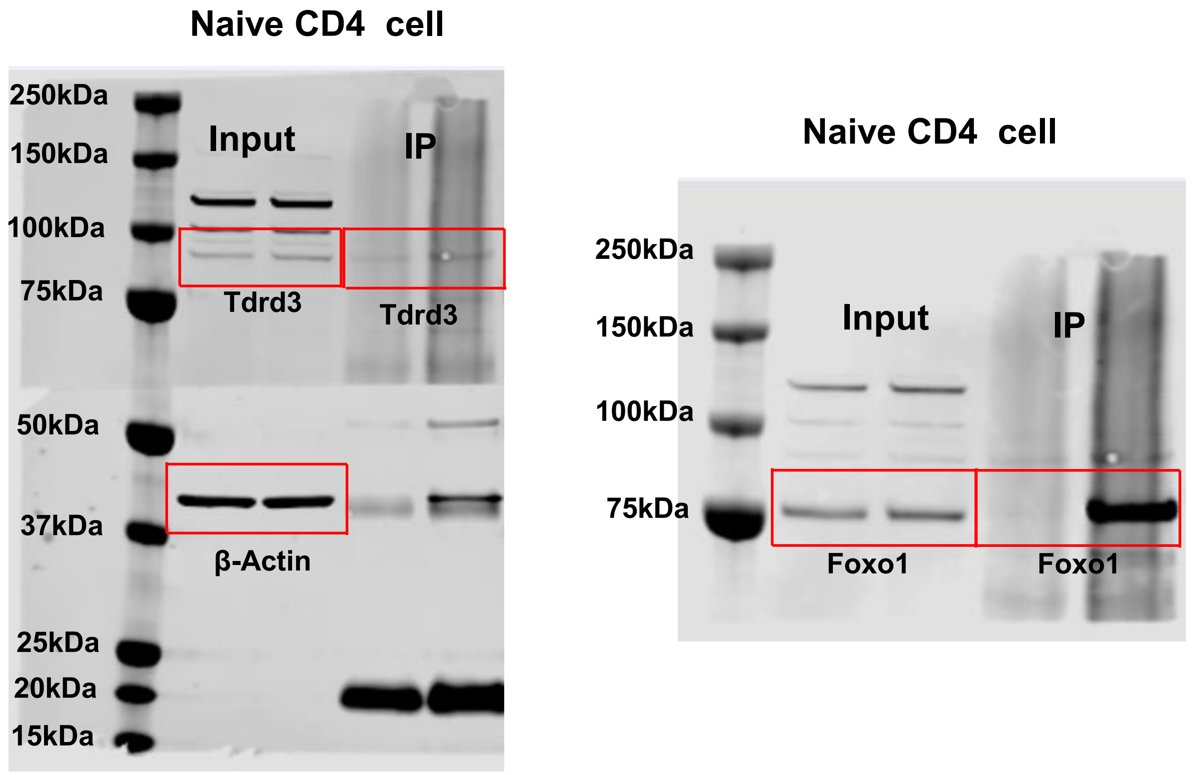

Fig. S7-continued

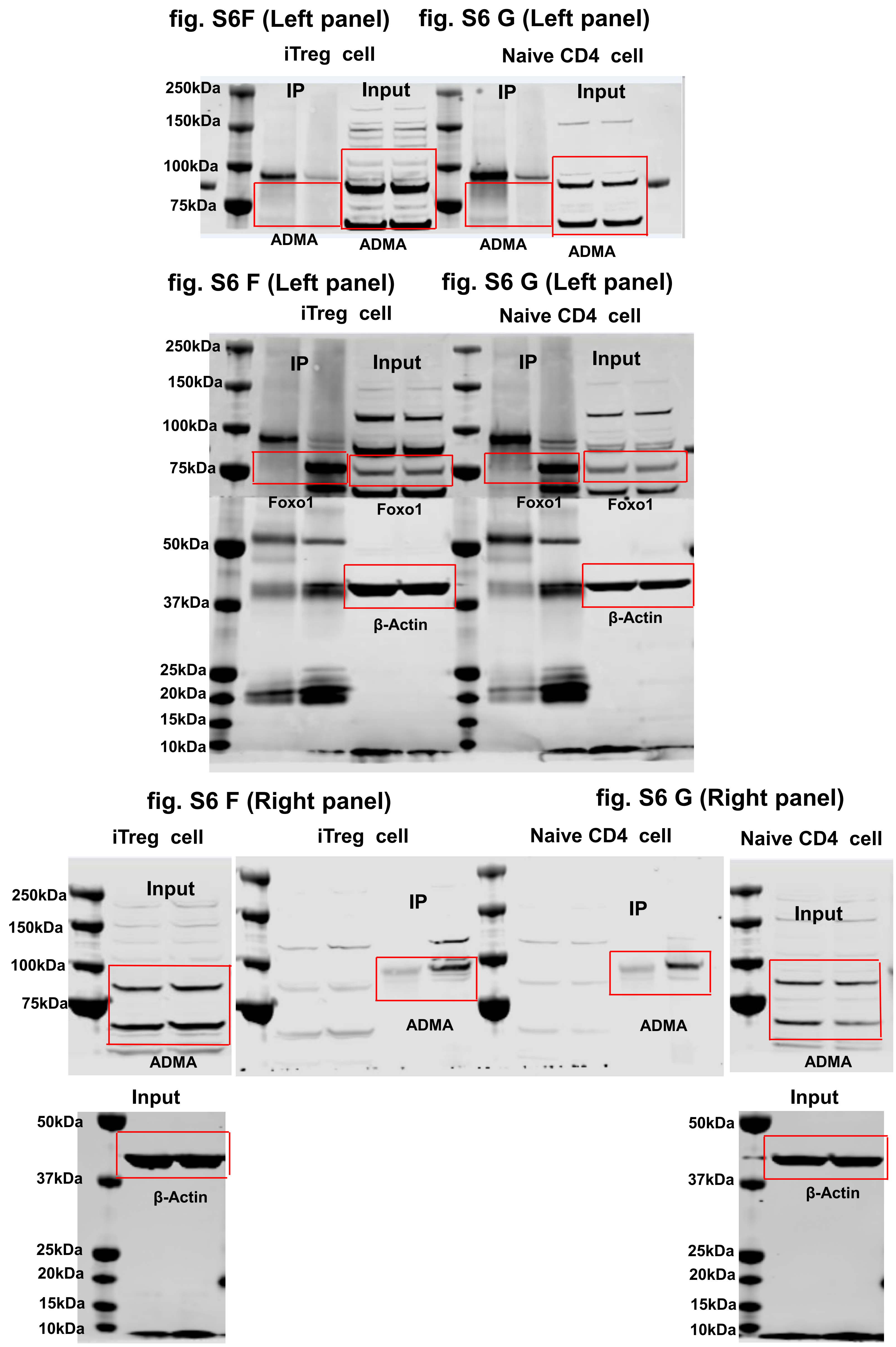

Fig. S7-continued

fig. S6 F (Right panel) fig. S6 G (Right panel)

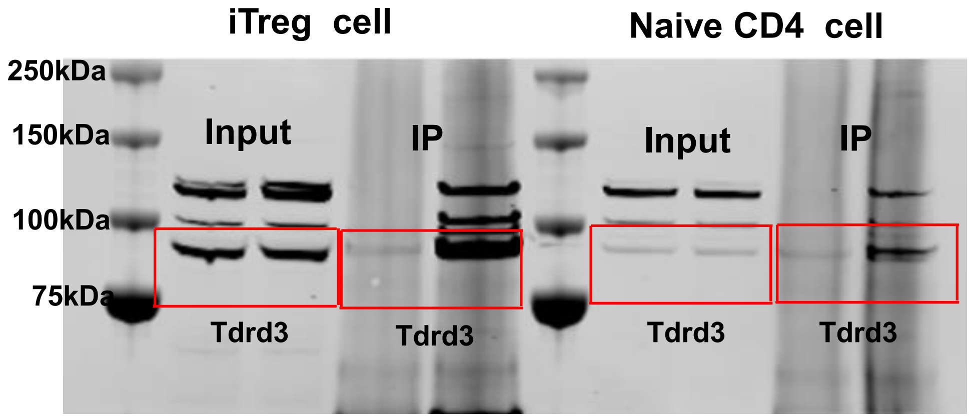

fig. S6 L

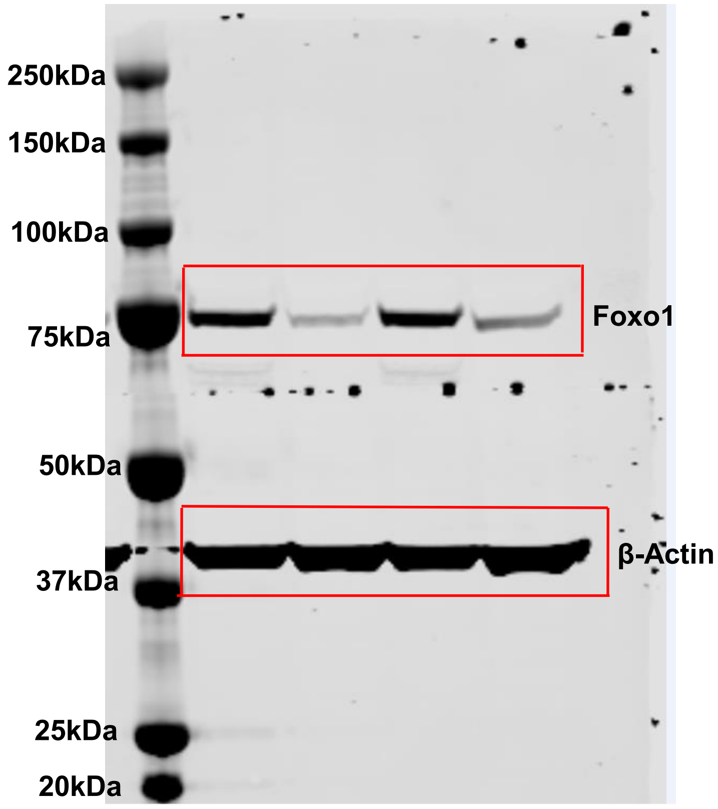

Table S1. List of primers sequences used in this study.

qPCR	Gene	Forward	Reverse
	Foxp3	CCCATCCCCAGGAGTCTTG	ACCATGACTAGGGGCACTGTA
	Tdrd3	AGCTTGCGATTCCTCGTTATC	CAACGGTGGAAAGGTAGAGAA
	Klf2	CACCTAAAGGCGCATCTGCGTA	GTGACCTGTGTGCTTTCGGTAG
	Myb	AGACCCCGACACAGCATCTA	CAGCAGCCCATCGTAGTCAT
	Actin	GGGAAATCGTGCGTGACAT	GTCAGGCAGCTCGTAGCTCTT
Chip-qPCR	Binding site	Forward	Reverse
	P1	GCCTCAACTCCCTTCTAGGC	CCAGGGAACGCTCTGTGAAA
	P2	TTTCACAGAGCGTTCCCTGG	CGAGGCTTTCCCGGTATCTC
	P3	GCTGTTGTGCCTGGTTTGTT	ATATAAGCCTGGCGGTGGTG
	P4	CAGGCTTATATACCGCGGCT	GGGACTGTGTGTGCTGTG
PCR	Binding site	Forward	Reverse
	P1	CAACTCCCTTCTAGGCAGGC	GGACCGATGCATGGGGA
	P2	TTCCAGGGGCTTGAGGCT	TCCCTATGGCGACGGCG
	P3-4	ATAGGGACGGTCGGGGG	GCAGGCACAGAGGGCC
CRISPR sgRNA		Sequence	
	sgP1#1	GGAGCATCGTAGCCCCCTTA	
	sgP1#2	GATTTCACAGAGCGTTCCCT	
	sgP2#1	ATGCATCGGTCCAGGTTTCC	
	sgP2#2	TATGGCGACGGCGTCAACAA	
	sgP3-4#1	CGACCGTCCCTATGGCGACG	
	sgP3-4#2	TATAGGCGCGGCAGGCACAG	
	sgNTC	GCGAGGTATTCGGCTCCGCG	
	sgKlf2	CGCGCGGGCTGAAGCTCGA	
	sgFoxo1#1	TACGCTGGCATGACGAATT	
	sgFoxo1#2	AATTCGGTCATGCCAGCGTA	
